# The role of Age, Gender, and Marital Status in prognosis for adults with depression: An Individual Patient Data Meta-analysis

**DOI:** 10.1017/S2045796021000342

**Published:** 2021-06-04

**Authors:** J. E. J. Buckman, R. Saunders, J. Stott, L-L. Arundell, C. O’Driscoll, M. R. Davies, T. C. Eley, S.D. Hollon, T. Kendrick, G. Ambler, Z. D. Cohen, E. Watkins, S. Gilbody, N. Wiles, D. Kessler, D. Richards, S. Brabyn, E. Littlewood, R. J. DeRubeis, G. Lewis, S. Pilling

**Affiliations:** 1 Centre for Outcomes Research and Effectiveness (CORE), Research Department of Clinical, Educational & Health Psychology, University College London, 1-19 Torrington Place, London WC1E 7HB; 2 iCope – Camden & Islington NHS Foundation Trust, St Pancras Hospital, London NW1 0PE; 3 Social, Genetic and Developmental Psychiatry Centre, Institute of Psychiatry, Psychology & Neuroscience, King’s College London; London SE5 8AF, UK; 4 Department of Psychology, Vanderbilt University, Nashville, TN 37240; 5 Primary Care, Population Sciences and Medical Education, Faculty of Medicine, University of Southampton, Southampton, SO16 5ST, UK; 6 Statistical Science, University College London, London, WC1E 7HB, UK; 7 Department of Psychiatry, University of California, Los Angeles, Los Angeles, CA, 90095, USA; 8 Department of Psychology, University of Exeter, Exeter, EX4 4QG, UK; 9 Department of Health Sciences, University of York, York, YO10 5DD, UK; 10 Centre for Academic Mental Health, Population Health Sciences, Bristol Medical School, University of Bristol, Oakfield House, Bristol BS8 2BN; 11 Centre for Academic Primary Care, Population Health Sciences, Bristol Medical School, University of Bristol, Canynge Hall, Bristol, UK; 12 Institute of Health Research, University of Exeter College of Medicine and Health, Exeter EX1 2LU, UK; 13 Department of Health and Caring Sciences, Western Norway University of Applied Sciences, Inndalsveien 28, 5063 Bergen, Norway; 14 School of Arts and Sciences, Department of Psychology, 425 S. University Avenue, Philadelphia PA, 19104-60185, USA; 15 MRC Clinical Trials Unit, Institute of Clinical Trials and Methodology, University College London, London, WC1V 6LJ, UK, Division of Psychiatry, University College London, London, W1T 7NF, UK; 16 Camden & Islington NHS Foundation Trust, 4 St Pancras Way, London, NW1 0PE

**Keywords:** Depression, Prognosis, Treatment Outcomes, Systematic Review, Individual Patient Data Meta-analysis

## Abstract

**Aims:**

To determine whether age, gender, and marital status are associated with prognosis for adults with depression who sought treatment in primary care.

**Methods:**

Medline, Embase, PsycINFO and Cochrane Central were searched from inception to 1^st^ December 2020 for randomised controlled trials (RCTs) of adults seeking treatment for depression from their general practitioners, that used the Revised Clinical Interview Schedule so that there was uniformity in the measurement of clinical prognostic factors, and that reported on age, gender and marital status. Individual participant data were gathered from all nine eligible RCTs (N=4864). Two-stage random-effects meta-analyses were conducted to ascertain the independent association between: i) age, ii) gender, and iii) marital status, and depressive symptoms at 3-4, 6-8, and 9-12 months post-baseline, and remission at 3-4 months. Risk of bias was evaluated using QUIPS and quality was assessed using GRADE. PROSPERO registration: CRD42019129512. Pre-registered protocol https://osf.io/e5zup/.

**Results:**

There was no evidence of an association between age and prognosis before or after adjusting for depressive ‘disorder characteristics’ that are associated with prognosis (symptom severity, durations of depression and anxiety, comorbid panic disorder, and a history of antidepressant treatment). Difference in mean depressive symptom score at 3-4 months post-baseline per-5-year increase in age =0(95%CI: -0.02 to 0.02). There was no evidence for a difference in prognoses for men and women at 3-4 months or 9-12 months post-baseline, but men had worse prognoses at 6-8 months (percentage difference in depressive symptoms for men compared to women: 15.08%(95%CI: 4.82 to 26.35)). However, this was largely driven by a single study that contributed data at 6-8 months and not the other time points. Further, there was little evidence for an association after adjusting for depressive ‘disorder characteristics’ and employment status (12.23%(-1.69 to 28.12)). Participants that were either single (percentage difference in depressive symptoms for single participants: 9.25%(95%CI: 2.78 to 16.13) or no longer married (8.02%(95%CI: 1.31 to 15.18)) had worse prognoses than those that were married, even after adjusting for depressive ‘disorder characteristics’ and all available confounders.

**Conclusion:**

Clinicians and researchers will continue to routinely record age and gender, but despite their importance for incidence and prevalence of depression, they appear to offer little information regarding prognosis. Patients that are single or no longer married may be expected to have slightly worse prognoses than those that are married. Ensuring this is recorded routinely alongside depressive ‘disorder characteristics’ in clinic may be important.

## Introduction

Patients and clinicians can benefit from prognostic information (Hayden et al., 2013). Such knowledge can inform routine clinical assessments and triages of patients seeking treatment for depression and might guide the collaborative treatment decision making process. This information does not help determine which treatment may be most likely to benefit any individual patient, but instead, informs considerations of the intensity, dose, or duration of treatment, the regularity of progress reviews, and referrals to specialist treatment centres (Buckman et al., 2021). This would be particularly true if the differences in prognoses for patients with one compared to another pre-treatment characteristic were of a clinically important magnitude (Button et al., 2015). Prognostic information is often wanted by patients and clinicians (Trusheim, Berndt and Douglas, 2007). However, prognostication for adults with depression is challenging as most prior studies have considered prognosis following one particular type of treatment, or in samples where either no treatment was given or details regarding any treatment(s) are unknown (Buckman et al., 2020; Buckman et al., 2021). As there is an approximate equivalence (on average) in the effectiveness of the most common types of treatment for depression (Menchetti et al., 2010; Cuijpers et al., 2013; Weitz et al., 2017; Cipriani et al., 2018), and a number of prognostic factors have been found to be associated with outcomes from one type of treatment but not others (Dodd et al., 2014; Chekroud et al., 2016; Nakabayashi, Hara and Minami, 2018; Buckman et al., 2021), there is a need to study prognosis regardless of the type of treatment received. Doing so would identify factors that are associated with prognosis in general, which would be informative for clinicians assessing and treating patients with depression in a breadth of settings. We call this prognosis independent of treatment. This form of prognosis has been largely overlooked in studies of depression (Buckman et al., 2020; Buckman et al., 2021). We recently found that a number of clinical factors were associated with prognosis independent of treatment, and that each contributed to explaining prognosis over and above depressive symptom severity (Buckman et al., 2021). We termed these depressive ‘disorder characteristics’. These were: the durations of depression and anxiety problems, comorbid panic disorder, and a history of antidepressant medication, each should be routinely assessed in clinic alongside depressive symptom severity. Whether other factors are similarly associated with prognosis and add incremental value to prognostic assessments is uncertain.

For decades it has been shown that age, gender, and marital status are associated with the prevalence of depression (Ensel, 1982; Kessler and Essex, 1982; Bebbington, 1987; Faravelli et al., 2013). Previous reviews of the relationship between these factors and post-treatment prognosis have found inconsistent results. For example, three reviews reported that older age was associated with worse prognosis (Cuijpers et al., 2018; Nakabayashi, Hara and Minami, 2018; Sockol, 2018); two found no such association (Johnsen and Friborg, 2015; Karyotaki et al., 2017); and one found that prognosis improved with increasing age (Noma et al., 2019). Similarly, four reviews reported that women had poorer prognoses than men (Carter et al., 2012; Dodd et al., 2014; Johnsen and Friborg, 2015; Noma et al., 2019), while four reported no such association (Karyotaki et al., 2017; Cuijpers et al., 2018; Nakabayashi, Hara and Minami, 2018; Schoemaker et al., 2018). Two reviews reported better prognoses for married individuals (Carter et al., 2012; Sockol, 2018) while a third found no evidence for this association (Karyotaki et al., 2017).

There are clear limitations, most of these past studies are not directly comparable as the reviews examined specific treatments only (e.g., duloxetine or Interpersonal Psychotherapy), and the sample settings were unclear or unstated. None of the past reviews presented results adjusted for the effects of multiple known clinical prognostic factors (Buckman et al., 2021). To do so accurately, for factors that differ at the individual patient level requires individual patient data (IPD) (Rothwell, 2005; Fisher, 2015) and only four of the past reviews had IPD. Two were not systematic reviews and made no adjustment for any clinical factors (Nakabayashi, Hara and Minami, 2018; Noma et al., 2019). Two others adjusted for baseline depressive symptom severity (Dodd et al., 2014; Karyotaki et al., 2017). The first (Dodd et al., 2014) made no adjustments for between-study effects and no assessments of either attrition or heterogeneity, limiting the robustness and interpretability of the findings. The second was an investigation of potential moderators of treatment effects and found high levels of heterogeneity that could not be explained (Karyotaki et al., 2017). Clarity on the association of these factors with prognosis is needed, particularly for patients initially seeking treatment in primary care as large proportions of adults are either initially screened and assessed or are wholly treated for depression in primary care settings (McManus et al., 2016; Olfson, Blanco and Marcus, 2016; Thornicroft et al., 2017).

Given these contrasting results, we aimed to determine whether age, gender, and marital status are associated with prognosis for adults with depression who sought treatment in primary care. We investigated these associations after accounting for the effect of known clinical prognostic factors, the depressive ‘disorder characteristics’ we identified previously (Buckman et al., 2021), and regardless of the type of treatment. In so doing, we aimed to determine whether these factors add incrementally to knowledge of prognosis at the point patients initially seek treatment.

## Methods

This systematic review with IPD meta-analysis is reported in accordance with the PRISMA-IPD statement (Stewart et al., 2015) (checklist in supplementary materials).

### Identification and selection of studies

A protocol for this study was pre-registered (https://osf.io/e5zup/). A general protocol for forming the Depression in General Practice (Dep-GP) individual patient data (IPD) dataset and the plan for analyzing those data (Buckman et al., 2020) and pre-registered methods for identifying studies are also available (PROSPERO: CRD42019129512 (01/04/2019)), and were reported in accordance with the PRISMA-P statement (Shamseer et al., 2015). Searches have been reported in accordance with PRISMA-S (Rethlefsen et al., 2021), with a brief description below and more details provided in Supplementary Materials.

Searches were conducted on Medline, Embase, International Pharmaceutical Abstracts, PsycINFO and Cochrane Central (searched from inception to 1^st^ December 2020), we also hand-searched reference lists, and contacted experts for unpublished or missed studies. Search terms included variations of phrases such as “depression” or “major depression”, “RCT” or “Randomized Controlled Trial”, and “CIS-R” or “Clinical Interview Schedule”. See Supplementary Table 1 for a full list and results of the searches.

A single reviewer (JB) screened titles and abstracts of potentially eligible studies, these were then read in full and judged against inclusion/exclusion criteria by two reviewers (JB and GL) with consultation with a third (SP) to resolve any uncertainties by consensus.

#### Inclusion & exclusion criteria

Studies were included if they were RCTs of adults (aged 16 or over) with unipolar depression, depressive symptoms significant enough to seek treatment, or a Revised Clinical Interview Schedule (CIS-R) (Lewis et al., 1992) score of ≥12 (the cut-off for common mental disorder); recruited from primary care; had at least one active treatment arm; used the CIS-R at baseline, and assessed marital status, age and gender at baseline.

Studies were excluded if they: included patients with depression secondary to a diagnosis of personality disorder, psychotic conditions, or neurological conditions; were studies of adults with bi-polar or psychotic depressions; were studies of children or adolescents; were feasibility studies; were studies of just one gender, one particular age group, or one marital status group only.

#### Data Extraction

The included studies are detailed in Table 1. Data were extracted for each study participant on all measures included in Table 2 and additional socio-demographics listed in Table 3 by the chief investigators or data managers of each study. Data were independently cleaned by two independent reviewers (JB and RS). Issues were resolved by consensus between four reviewers (JB, RS, GL and SP).

#### Data Integrity Checks

Integrity of all baseline and endpoint data for each study was checked with the study team and against publications from each study. We removed two participants from IPCRESS and one from PANDA due to them missing data on over 75% of baseline variables. There were also data available on 36 more participants in ITAS than reported in the publication about that study.

### Ethical Considerations

All studies were granted NHS Research ethical approvals and all participants gave informed consent, see Supplementary Table 2. No additional ethical approval was required for this study: HRA reference 712/86/32/81.

### Measures

See Table 2 for details.

### Data analysis plan

The pre-registered analysis plan (https://osf.io/e5zup/) is outlined below It builds on the plan outlined in our general protocol (Buckman et al., 2020).

#### Primary outcomes

Depressive symptoms at 3-4 months post-baseline were captured in two ways. 1) z-score (standardised mean) of the scores on the depressive symptom measures (BDI-II and PHQ-9) used at 3-4 months post-baseline within each study (see Table 1). The score at 3-4 months was divided by the standard deviation for that measure calculated at 3-4 months. 2) The logarithm of depression scale scores irrespective of the measure used. Exponentiation of the regression coefficient provides an estimate of the percentage difference in symptoms.

It was expected that these methods would give similar results but the log outcome might have greater utility as percentage differences are more easily understood and do not require division by standard deviation estimates.

#### Secondary outcomes

1. Remission on the primary depression measure in each study at 3-4 months post-baseline (a score of <10 on the BDI-II or PHQ-9, whichever was the primary measure collected at that time point in each study).
2. Depressive symptoms at 6-8 months post-baseline, as above but also including the GHQ-12, captured with i) the z-score calculated using the mean and standard deviation for the scores at 3-4 months, and ii) the logarithm of scores at 6-8 months.
3. Depressive symptoms at 9-12 months post-baseline, as above, captured with i) the z-score calculated using the mean and standard deviation for the scores at 3-4 months, and ii) the logarithm of scores at 9-12 months.

#### Prognostic indicators under consideration

1. Age was considered both as a continuous variable per 5-year increase, and in age groups: i) 16-29 ii) 30-39; ii) 40-49; iii) 50-59; iv) ≥60 years old.
2. Gender was taken from a question asking participants for their self-reported gender at baseline, in all included studies response options were binary. This was commonplace in studies at the time, it does not capture the appropriate range of gender identities, neither does it capture differences between gender and sex assigned at birth.
3. Marital Status was initially recorded in five categories: 1) married or cohabiting (living with partner); 2) single (never married), 3) separated, 4) divorced, 5) widowed. However, some of the studies had very few participants that were divorced (n=0 in ITAS) and widowed (n=4 in GENPOD, n=1 in IPCRESS and n=6 in TREAD). Therefore, marital status was recoded into three categories to meet power requirements by combining categories 3-5 above in a new category “no longer married”.

#### Confounders and covariates

Data on some potential confounders were available in all studies whereas data on others were not (i.e., systematically missing). Following our protocol, we adjusted for the following in separate models of each outcome:

1. Depressive ‘disorder characteristics’: depressive symptom severity, the duration of the current depressive episode, mean duration of anxiety problems, comorbid panic disorder, and a history of antidepressant treatment
2. Age, gender and marital status (excluding whichever of these was already included in the model as the potential prognostic factor)
3. Employment Status

In sensitivity analyses we then added each of the systematically missing variables one at a time:

1. Housing Status (available in eight studies)
2. Long-term physical health condition status (yes/no; available in eight studies)
3. Highest level of educational attainment (available in seven studies)
4. Financial wellbeing (available in six studies)
5. Social support (available in six studies)

In addition, all models were adjusted for treatment allocation by assigning each of the randomized groups across the studies a different category in a single categorical variable and including this variable in all models. This meant that associations between the prognostic factors and outcomes could be investigated having adjusted for the effects of the randomized treatments. It also meant that all models accounted for clustering effects at the study-level.

#### Data handling and data management

Details of the pre-processing stages and handling of missing data are given in the original protocol (Buckman et al., 2020). Multiple imputation with chained equations was used with baseline and outcome variables to generate 50 imputed datasets for each study. Systematically missing variables were not imputed.

#### Primary analyses

Two-stage DerSimonian and Laird random effects meta-analyses were conducted for each prognostic factor (each fitted into separate models) for each outcome variable listed above, using the “admetan” package (Fisher, 2015) in Stata 16 (StataCorp, 2019). Linear models were fitted for the z-score and log outcomes and logistic models were fitted for remission. The degree of heterogeneity was assessed using prediction intervals and its impact assessed using the I^2^ statistic (Higgins et al., 2003).

### Risk of Bias

Two reviewers (JB & RS) conducted independent risk of bias assessments using the Quality in Prognosis Studies (QUIPS) (Hayden et al., 2013) and the quality of evidence for each prognostic indicator was assessed using the GRADE framework (Guyatt et al., 2008). Disagreements were resolved by consensus with two additional reviewers (GL and SP).

### Sensitivity analyses

In addition to those listed above sensitivity analyses were conducted if there was considerable heterogeneity (Higgins and Green, 2011) either from inspection of the forest plots or if I^2^ was 75% or above, or if either study quality was low or risk of bias was high, removing the study contributing most to the heterogeneity/low quality/high risk of bias.

## Results

### Characteristics of the included studies

**Figure 1:**
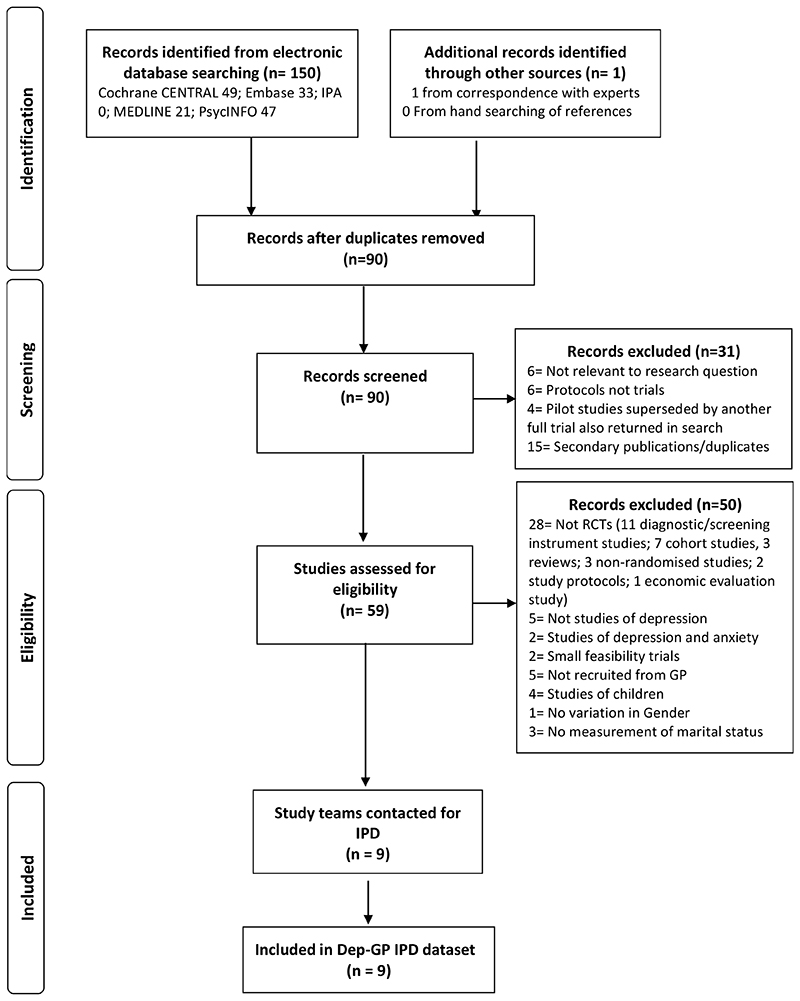
Flow of studies through selection process for IPD meta-analysis

In total nine RCTs met the inclusion criteria. IPD from all 4864 participants formed the present dataset, see Figure 1.

### Quality assessments and Risk of Bias

Overall, the risk of bias was low, and quality was high in all studies, so no sensitivity analyses were required in relation to these, although half of the studies had a moderate risk of bias related to attrition, see Supplementary Tables 3-4. There was near perfect agreement between the reviewers, with interrater reliability (Cohen’s Kappa) k=0.96 for QUIPS and k=1.00 for GRADE.

### Descriptive Statistics

Across all nine studies, age ranged between 16-84 years old at baseline with a mean age of approximately 42 years, 80% of the sample were aged between 23-61 years old. Approximately 67% of the participants were women, half of participants were married or cohabiting, approximately 30% were single and 20% no longer married (Table 3).

### Association between age and prognosis independent of treatment

Overall, there was no evidence of an association between age and prognosis at 3-4 months post-baseline. This was the case when age was modelled as a continuous variable, an ordinal variable of age groups, comparing each age group to those aged 16-29 years old (see Table 4), and with age modelled with a quadratic term (p=0.233). There was also no evidence of an association between age and prognosis at 6-8 months (Supplementary Tables 7-8), or 9-12 months (Supplementary Tables 9-10).

### Associations between gender and prognosis independent of treatment

There was no evidence for a difference in prognosis between men and women at 3-4 months (Tables 4-5) or 9-12 months (Supplementary Tables 9-10) post-baseline, but there was evidence that men had worse prognoses at 6-8 months (percentage difference in depressive symptoms for men compared to women: 15.08%(95%CI: 4.82 to 26.35), see Supplementary Table 8). However, this was attenuated when adjusting for socio-demographic factors: age, marital status and employment, 12.23%(-1.69 to 28.12).

### Associations between marital status and prognosis independent of treatment

There was some evidence of an association between marital status and prognosis at 3-4 months (Tables 4-5). Participants who were single (difference in mean depressive symptom score at 3-4 months (0.22(95%CI: 0.15 to 0.30)) and those who were no longer married (0.28(95%CI: 0.20 to 0.37)), had more depressive symptoms at 3-4 months post-baseline on average, compared to married participants. After adjusting for the ‘disorder characteristics’ and socio-demographic factors there was still evidence that single or no longer married participants had worse prognoses than those that were married, but the strength of the associations was somewhat attenuated (see Tables 4-5). There were similar effects at 6-8 months post-baseline although confidence intervals included zero when controlling for employment status in addition to all other confounders with the log outcome (Supplementary Tables 7-8). Further, when comparing just those participants who reported being single to those who reported being married, there was a lack of evidence for an association with prognosis at 6-8 months post-baseline after adjusting for depressive ‘disorder characteristics’. At 9-12 months there was no evidence for an association between marital status and prognosis after adjusting for the depressive ‘disorder characteristics’ (Supplementary Tables 9-10).

### Sensitivity analyses

Sensitivity analyses including systematically missing confounding factors did not substantively change our conclusions (see Supplementary tables 12-18), nor did analyses that removed studies due to high levels of heterogeneity (Supplementary Table 19).

## Discussion

This study investigated the associations between age, gender, and marital status, with the prognosis of depression for adults that sought treatment in primary care, regardless of the type of treatment they were given. We found no evidence for an association between age and prognosis at 3-4, 6-8 or 9-12 months post-baseline and no evidence that gender was associated with prognosis after adjusting for clinical prognostic factors and employment status. There was evidence that single, and either separated, divorced, or widowed patients (those no longer married) had worse prognoses than married patients at 3-4 months. However, the effect was weaker after adjusting for clinical prognostic factors and employment status.

Our recent review of systematic reviews and meta-analyses found inconsistent and at times contradictory findings regarding the associations between age, gender, marital status and prognosis for depressed patients (Buckman et al., 2021). Our findings suggest that the pattern of associations found in those past reviews may have been due to chance, or the specific features of each review. With greater power to detect effects due to having a large individual patient data sample here, and additional robustness by adjusting for a range of treatments and for important clinical and demographic prognostic factors, we found no overall association between either age or gender, and prognosis. Although we found the that men appeared to have worse prognoses than women at 6-8 months this was attributable to a single large study that contributed data at the 6-8 month endpoint and not at 3-4 or 9-12 months, and the association was minimal after adjusting for clinical and demographic covariates. There had been limited evidence for an association between marital status and prognosis in prior reviews, here we did find evidence of such an association in our data (married patients did better with treatment). On the basis of our findings here it is unlikely that knowledge of patients’ self-reported marital statuses will inform clinically meaningful differences in prognosis alone, particularly after accounting for depressive ‘disorder characteristics’ and employment status (Button et al., 2015). However, it may be important to consider in conjunction with those other factors. The strength of the association with prognosis likely lies somewhere between the prognostic effects found for a history of antidepressant medication and comorbid panic disorder (Buckman et al., 2021).

### Strengths and Limitations

To our knowledge, this is the first study to use a large individual patient dataset formed from a number of RCTs to consider the prognostic status of age, gender, and marital status across different types of treatment, bringing greater precision to estimates of these associations than in past studies. In contrast to past reviews, we selected studies that included treatment seeking adults with depression recruited in primary care settings, as this is a very common route into treatment (McManus et al., 2016; Thornicroft et al., 2017). This may have limited the number of studies found to meet inclusion criteria for this review, but has the advantage of ensuring there is a minimum population for whom our findings may be generalizable. Further, as many of the randomized treatments here are commonly used in other settings, our findings may be informative for clinicians assessing depressed patients in other settings where there are multiple treatment options.

In addition, our inclusion criteria specified such that all studies used the same measure to determine diagnosis, assess baseline symptoms, and the depressive ‘disorder characteristics’ confounders, minimizing bias in harmonizing the data across studies. This reduced the potential pool of studies that might have been included here. However, as IPD data were available for all studies that met these criteria we did not introduce a common source of selection bias that can occur when only a subset of trials provide IPD. It is noteworthy that from our preliminary searches only two other studies (Hegerl et al., 2010; Perroud et al., 2012) (totalling n=612 patients) might potentially have met all other inclusion criteria and used other comprehensive measures of depressive and anxiety symptoms and disorders at baseline. An alternative approach might have been taken by not specifying which measure was used at baseline but instead only specifying that all of the ‘disorder characteristics’ noted to be associated with prognosis in our previous study should have been assessed. It is noteworthy though that many studies that were excluded contained no comprehensive measure of anxiety disorder symptoms or diagnoses and so most would not have assessed for panic disorder, thus would have failed to meet inclusion criteria in this way too.

We used robust methods whereby data were extracted, cleaned, and checked by multiple reviewers (Buscemi et al., 2006), but only a one reviewer screened the initial titles and abstracts of the studies returned in the searches which may have introduced additional bias. Although adjustments were made for a number of potential confounders, including clinical, demographic, and socio-economic variables, we cannot rule out residual confounding. Further, it is possible that adjusting for baseline depressive severity may have led to underestimating the effects of marital status on prognosis, as higher levels of baseline severity might be associated with a greater likelihood of separation, divorce, or of being single. In addition, using a standardized outcome is a method that has been criticized but the results using the z-score outcome were similar to those with the log outcome and the secondary and sensitivity outcomes, suggesting the use of the standardized outcome metric did not unduly affect the results. The included studies were conducted at such a time when only two categories of gender were routinely assessed and did not give participants the option to identify with non-binary gender identities. Two participants had no gender data recorded; it is not clear whether they would have chosen another gender identity were other categories available to them.

### Implications and Conclusions

Clinicians and researchers will continue to routinely assess for age and gender however the value of including information on these factors when considering prognosis is likely to be very limited. On the basis of these findings we might conclude that although age and gender are important for the incidence and prevalence of depression (Kessler et al., 2005), they appear to not be important for prognosis with treatment. Patients that are married appear to have better prognoses than patients that are single or no longer married. On its own, marital status is unlikely to inform a clinically important difference in prognosis after accounting for the severity of depressive illness, captured by the depressive ‘disorder characteristics’. However, as the magnitude of effects was similar to those of a number of other clinical prognostic factors, it may be equally important to assess for marital status in clinic and to include it in predictive models of prognosis. We were unable to investigate why married people have better prognoses. It is noteworthy that when adjusting for housing status, financial wellbeing and social support (in sensitivity analyses) the associations were weaker, so married patients might differ from those that are not married in these social domain, and these in turn may be associated with prognosis.

## Supplementary Material

Supplementary Material

## Figures and Tables

**Table 1:** Description of included studies

		Inclusion criteria	Age	Gender	Marital Status	T0 Depressive Severity	Remission		Outcome Measure
Study	N		Mean(SD)	%Female	% married, single, no longer married	Mean(SD)	% at 3-4 months	Interventions	Primary (additional)
CADET (Richards et al., 2013)	527	Adults ≥18, ICD-10 Depressive Episode	44.4(13.2)	72%	41.4% married; 30.2% single; 28.5% no longer married	PHQ-9=17.7(5.1)	41%	Collaborative Care vs TAU	PHQ-9
COBALT (Wiles et al., 2013)	469	Adults 18-75 with treatment resistant depression, scoring ≥14 BDI-II	49.6(11.7)	72%	52.9% married; 19.0% single; 28.1% no longer married	BDI-II=31.8(10.7)	34%	CBT+TAU vs TAU	BDI-II (PHQ-9)
GENPOD (Wiles et al., 2012)	601	Adults 18-74 with depressive episode	38.8(12.4)	68%	52.6% married; 29.1% single; 18.3% no longer married	BDI-II=33.7(9.7)	41%	Citalopram vs Reboxetine	BDI-II
IPCRESS (Kessler et al., 2009)	295	Adults scoring ≥14 BDI-II and GP confirmed diagnosis of depression	34.9(11.6)	68%	36.6% married; 47.8% single; 15.6% no longer married	BDI-II=33.2(8.8)	34%	iCBT+TAU vs TAU + waiting list for iCBT	BDI-II
ITAS (Thomas et al., 2004)	798	Adults ≥16, scored ≥12 on CIS-R	43.2(14.8)	68%	58.7% married; 22.4% single; 18.9% no longer married	GHQ=7.7(3.2)	N/A; at 6-8 months 46%	Recommendation + TAU vs TAU	GHQ-12
MIR (Kessler et al., 2018)	480	Adults ≥18 taking SSRIs or SNRIs at adequate dose for≥ 6 weeks, and scored ≥14 on BDI-II	50.7(13.2)	69%	59.4% married; 19.6% single; 21.0% no longer married	BDI-II=31.1(9.9)	30%	Mirtazapine vs Placebo	BDI-II (PHQ-9)
PANDA (Lewis et al., 2019)	652	Adults presenting with low mood or depression to GP in last 2 years, free of ADM for 8 weeks up to baseline	39.7(15.0)	59%	39.1% married; 45.4% single; 15.5% no longer married	BDI-II=23.9(10.3)	69%	Sertraline vs Placebo	PHQ-9 (BDI-II)
REEACT (Gilbody et al., 2015)	685	Adults with PHQ-9≥10 presenting to GP with depression	39.9(12.7)	67%	50.9% married; 33.5% single; 15.6% no longer married	PHQ-9=16.7(4.3)	53%	Moodgym vs Beating the Blues vs TAU	PHQ-9
TREAD (Chalder et al., 2012)	361	Adults 18-69 who met diagnostic criteria for MDD and scored ≥14 on BDI-II	39.8(12.6)	66%	46.3% married; 32.1% single; 21.6% no longer married	BDI-II=32.1(9.2)	35%	Physical Activity + TAU vs TAU	BDI-II

Abbreviations: ADM – antidepressant medication; BDI-II – Beck Depression Inventory; EPDS – Edinburgh Postnatal Depression Scale; GHQ-12 – General Health Questionnaire 12 item version; HADS-D – Hospital Anxiety and Depression Scale – depression subscale; iCBT (internet based therapist delivered cognitive behavioural therapy); MDD – Major Depressive Disorder; T0-Baseline; TAU – treatment as usual; TCA – tricyclic antidepressant

**Table 2:** Measures used across the studies of the Dep-GP IPD dataset meeting inclusion criteria for the present study

Measure	Details	Scores and Cut-offs for Remission
The CIS-R(Lewis et al., 1992)	Consists of 14 symptom subsections scored 0-4 covering core features of depression, depressive thoughts (scored 0-5), fatigue, concentration/forgetfulness, and sleep, generalized anxiety, worry, irritability, obsessions, compulsions, health anxiety, somatic concerns, phobic anxiety (split into agoraphobia, social phobia, and specific phobia), and panic. A final section measures general health, impairment and weight change.	The total score ranges from 0-57 with a cut-off of ≥12 used to indicate likely common mental disorder, primary and secondary diagnoses using ICD-10 criteria are given as are binary indictors of diagnosis for all the disorders assessed. The duration of each type of problem is also assessed for the present episode (or subsyndromal episode) up to the point of completing the CIS-R. Duration items are measured in five categories: 1) less than two weeks; 2) between two weeks and six months; 3) between six months and one year; 4) between one and two years; and 5) more than two years.
Beck Depression Inventory 2^nd^ Edition (BDI-II)(Beck, Steer and Brown, 1996)	Consists of 21 items to assess depressive symptoms, each item is scored 0-3.	There is a maximum score obtainable of 63, and a cut-off of ≥10 is used indicate significant symptoms of depression, scores of <10 are therefore used to indicate remission in those that were previously depressed/scored ≥10.
Patient Health Questionnaire 9-item version (PHQ-9)(Kroenke, Spitzer and Williams, 2001)	This is a depression screening measure, with respondents asked to rate how often they have been bothered by each of the nine symptom items over the preceding two weeks. Each item is scored 0-3	There is a maximum score of 27 with a cut-off of ≥10 is used to indicate “caseness” for depression, a score of 9 or below for those that were previously depressed is therefore considered to indicate remission
General Health Questionnaire (12-item version) (GHQ-12)(Goldberg, 1992)	Consists of 12 items related to present and recent health over the “few weeks” prior to completion. Each item is related to depression or generalized anxiety, they are scored 0-0-1-1 for the four response options.	A cut-off of ≥2 is used to indicate the likely presence of common mental disorder, and so scores of <2 for those formally scoring above this would be considered to indicate remission
Social Support Scale-adapted by authors of RCTs (Kessler et al., 2009) included in this IPD by adding one item to the Health and Lifestyles Survey Social Support Measure(Cox et al., 1987)	*An 8-item instrument (the first seven of which are from the Health and Lifestyles Survey) assessing the degree to which participants rated the* social support of their friends and family in each of the following domains: 1) being accepted for who one is; 2) feeling cared about; 3) feeling loved; 4) feeling important to them; 5) being able to rely on them; 6) feeling well supported and encouraged by them; 7) being made to feel happy by them; and 8) feeling able to talk to them whenever one might like. *Items are scored 1-3, with total scores ranging from 8-24; higher scores indicate higher levels of perceived social support. The authors of the Health and Lifestyles Survey suggested the maximum score for social support (which was 21 on that scale) indicated ‘no lack of social support’, scores between 18-20 indicated a ‘moderate lack of social support’, and scores of 17 or below indicated a ‘severe lack of social support’*.	N/A

CIS-R was used in all studies n=4864. BDI-II was used in 6 studies (COBALT, GENPOD, IPCRESS, MIR, PANDA, & TREAD), n=2858 ; PHQ-9 was used in 5 studies (CADET, COBALT, MIR, PANDA, & REEACT) n=2807; GHQ was used in ITAS only n =796; and the Social Support Scale was used in 6 studies (COBALT, GENPOD, IPCRESS, MIR, PANDA, & TREAD) n =2858

**Table 3:** Baseline characteristics of study sample using observed data only

Self-reported Baseline Characteristics	Factor	N(%), or N and Mean(SD)
**Total Sample Size (N)**		4864
**Age in years**		N: 4864, Mean(SD): 42.45(13.99)
**Age 16-29 years old**		1079(22.18%)
**Age 30-39 years old**		1043(21.44%)
**Age 40-49 years old**		1208(24.84%)
**Age 50-59 years old**		919(18.89%)
**Age 60+ years old**		615(12.64%)
**Gender**	Female	3279(67.41%)
	Male	1583(32.55%)
	Missing	2(0.04%)
**Marital Status**	Married/cohabiting	2412(49.59%)
	Single	1477(30.37%)
	No longer married	975(20.05%)
**Employment status**	Employed	2713(55.81%.)
	Not seeking employment	1199(24.67%)
	Unemployed	949(19.52%)
	Missing	3(0.06%)
**Housing Status**	Home Owner	2148(48.92%)
	Tenant	1677(38.19%)
	Other	566(12.89%)
	Missing	473(9.72%)
**Financial Wellbeing**	Doing OK financially	1537(42.14%)
	Just about getting by	1171(32.11%0
	Struggling Financially	939(25.75%)
	Missing	1217(25.02%)
**Highest level of educational attainment**	Degree or above	959(28.02%)
	A-level of Diplomas	905(26.44%)
	GCSE	1016(29.68%)
	None or Other	543(15.86%)
	Missing	1441(29.63%)
**Long term Physical Health Condition**	No	3244(73.81%)
	Yes	1151(26.19%)
	Missing	469(9.64%)
**Social Support Scale Score**		N: 2858, Mean(SD): 20.25(3.85)
**Past Antidepressant use**	No	1241(25.53%)
	Yes	3620(74.47%)
**CIS-R durations**	Depression	N: 4864, Mean(SD): 3.32(1.37)
	Average Anxiety Duration	N: 4813. Mean(SD): 2.05(0.98)
**Comorbid panic disorder**	No	4439(91.26%)
	Yes	425(8.74%)
**Baseline BDI-II score**		N: 2858, Mean(SD): 30.44(10.53)
**Baseline PHQ-9 score**		N: 2812, Mean(SD): 15.71(5.65)
**Baseline GHQ-12 score**		N: 795, Mean(SD): 7.69(3.22)
**Attrition at 3-4 months**	No	3411(70.13%)
	Yes	658(13.53%)
	Not Applicable	795(16.34%)
**BDI-II score 3-4 months**		N:1918, Mean(SD): 16.07(11.99)
**PHQ-9 score 3-4 months**		N:2393, Mean(SD): 10.28(6.65)
**Remission 3-4 months**	No	1928(56.57%)
	Yes	1480(43.43%)
**BDI-II score 6-8 months**		N:1236, Mean(SD): 18.64(13.44)
**PHQ-9 score 6-8 months**		N:814, Mean(SD): 10.33(6.78)
**GHQ-12 score 6-8 months**		N:585, Mean(SD): 3.80(4.10)
**Attrition at 6-8 months**	No	1236(25.41%)
	Yes	369(7.59%)
	Not Applicable	3259(67.00%)
**BDI-II score 9-12 months**		N:1028, Mean(SD): 16.78(12.87)
**PHQ-9 score 9-12 months**		N:1764, Mean(SD): 9.51(6.71)
**Attrition at 9-12 months**	No	2005(41.22%)
	Yes	516(10.61%)
	Not Applicable	2343(48.17%)

**Table 4:** Primary Outcomes Difference in Z-score of depressive symptoms (“mean difference”) at 3-4 months post-baseline per unit increase in baseline prognostic indicator

	Adjusted for treatment			Additionally adjusted for depressive severity and ‘disorder characteristics’			Additionally adjusted for age, gender, and marital status			Additionally adjusted for age, gender, marital status, and employment status		
Baseline Variable	Z-score of depressive symptoms	Number of Studies	Heterogeneity	Z-score of depressive symptoms	S Number of Studies	Heterogeneity	Z-score of depressive symptoms	Number of Studies	Heterogeneity	Z-score of depressive symptoms	Number of Studies	Heterogeneity
	Mean difference (95%CI)	K	I^2^	Mean difference (95%CI)	K	I^2^	Mean difference (95%CI)	K	I^2^	Mean difference (95%CI)	K	I^2^
Age (per 5 year increase)	-0.01(-0.04 to 0.01)	8	73	0(-0.02 to 0.02)	8	62	-0.06(-0.16 to 0.04)	8	0	-0.06(-0.16 to 0.04)	8	0
Age Group⸷	-0.03(-0.08 to 0.02)	8	74	0.01(-0.03 to 0.05)	8	65	-0.04(-0.13 to 0.05)	8	0	-0.05(-0.14 to 0.04)	8	0
Age (16-29 - reference)	0			0			0			0		
Age (30-39)	-0.07(-0.17 to 0.03)	8	0	-0.03(-0.13 to 0.07)	8	13	0.00(-0.11 to 0.11)	8	7	-0.01(-0.11 to 0.09)	8	0
Age (40-49)	-0.00(-0.21 to 0.21)	8	78	0.01(-0.13 to 0.16)	8	55	0.04(-0.10 to 0.19)	8	47	0.03(-0.12 to 0.18)	8	49
Age (50-59)	0.03(-0.15 to 0.16)	8	45	0.07(-0.08 to 0.21)	8	49	0.08(-0.06 to 0.23)	8	33	0.06(-0.08 to 0.19)	8	25
Age (60+)	-0.14(-0.35 to 0.06)	8	63	0.05(-0.11 to 0.20)	8	39	0.06(-0.11 to 0.24)	8	40	0.05(-0.13 to 0.23)	8	46
Gender (women - reference)	0			0			0			0		
Gender (men)	0.01(-0.06 to 0.09)	8	7	0.05(-0.02 to 0.11)	8	0	0.05(-0.02 to 0.11)	8	0	0.03(-0.03 to 0.09)	8	0
Marital Status⸷	0.15(0.11 to 0.2)	8	0	0.1(0.06 to 0.14)	8	0	0.1(0.06 to 0.14)	8	0	0.08(0.04 to 0.12)	8	0
Married (reference)	0			0			0			0		
Single	0.22(0.15 to 0.30)	8	0	0.14(0.07 to 0.21)	8	2	0.15(0.08 to 0.23)	8	0	0.10(0.03 to 0.18)	8	0
No longer Married	0.28(0.20 to 0.37)	8	0	0.18(0.10 to 0.26)	8	0	0.18(0.10 to 0.26)	8	0	0.13(0.05 to 0.21)	8	0

Note

⸷ Association for ordinal variables is per category increase from first category shown below the variable down to the last (i.e. married to single, single to no longer married).’Disorder characteristics’ adjusted for are: baseline BDI-II score, average anxiety duration, depression duration, comorbid panic disorder, and history of antidepressant treatment

**Table 5:** Percentage difference (“% difference”) in depressive symptom scale scores at 3-4 months post-baseline per unit increase in baseline prognostic indicator

	Adjusted for treatment			Additionally adjusted for depressive severity and ‘disorder characteristics’			Additionally adjusted for age, gender, and marital status			Additionally adjusted for age, gender, marital status, and employment status		
Baseline Variable	Percentage difference in depressive symptoms	Number of Studies	Heterogeneity	Percentage difference in depressive symptoms	Number of Studies	Heterogeneity	Percentage difference in depressive symptoms	Number of Studies	Heterogeneity	Percentage difference in depressive symptoms	Number of Studies	Heterogeneity
	% difference (95%CI)	K	I^2^	% difference (95%CI)	K	I^2^	% difference (95%CI)	K	I^2^	% difference (95%CI)	K	I^2^
Age (per 5 year increase)	-0.75(-2.61 to 1.14)	8	73	0.45(-1.27 to 2.20)	8	68	-2.10(-9.68 to 6.11)	8	0	-2.47(-9.97 to 5.66)	8	0
Age Group⸷	-1.45(-5.26 to 2.51)	8	73	0.97(-2.7 to 4.78)	8	69	-3.23(-10.19 to 4.27)	8	0	-4.03(-10.87 to 3.32)	8	0
Age (16-29 - reference)	0			0			0			0		
Age (30-39)	-8.41(-16.62 to 0.60)	8	19	-6.06(-14.13 to 2.78)	8	18	-3.45(-11.46 to 5.27)	8	6	-3.80(-11.60 to 4.70)	8	0
Age (40-49)	-3.08(-16.93 to 13.08)	8	73	-1.13(-10.69 to 9.46)	8	40	2.29(-7.49 to 13.11)	8	28	2.09(-7.82 to 13.06)	8	29
Age (50-59)	-0.58(-11.03 to 11.08)	8	40	3.57(-9.17 to 18.11)	8	57	6.24(-6.25 to 20.38)	8	43	4.50(-7.40 to 17.94)	8	38
Age (60+)	-8.92(-24.30 to 9.59)	8	71	3.47(-11.23 to 20.62)	8	58	6.47(-9.63 to 25.43)	8	56	4.04(-12.67 to 23.94)	8	60
Gender (women-reference)	0			0			0			0		
Gender (men)	2.9(-2.53 to 8.63)	8	0	4.13(-1.1 to 9.63)	8	0	4.33(-1.03 to 9.99)	8	4	2.61(-3.02 to 8.56)	8	15
Marital Status⸷	10.07(6.61 to 13.64)	8	0	6.82(3.64 to 10.1)	8	0	6.81(3.61 to 10.12)	8	0	4.85(1.61 to 8.19)	8	3
Married (reference)	0			0			0			0		
Single	18.12(11.34 to 25.31)	8	0	12.02(5.95 to 18.44)	8	0	13.76(7.10 to 20.83)	8	0	9.25(2.78 to 16.13)	8	0
No longer Married	19.17(11.64 to 27.21)	8	0	12.72(5.92 to 19.94)	8	0	11.97(5.10 to 19.29)	8	0	8.02(1.31 to 15.18)	8	0

Note

⸷ Association for ordinal variables is per category increase from first category shown below the variable down to the last (i.e. married to single, single to no longer married). ‘Disorder characteristics’ adjusted for are: baseline BDI-II score, average anxiety duration, depression duration, comorbid panic disorder, and history of antidepressant treatment

## Data Availability

Requests for sharing of the IPD used in this study can be made to the corresponding author, any sharing of data will be subject to obtaining appropriate agreements from the chief investigators or data custodians for each individual trial dataset used here.
