# Supplementary material for: The role of Age, Gender, and Marital Status in prognosis for adults with depression: An Individual Patient Data Meta-analysis
Source: Epidemiol Psychiatr Sci. Author manuscript; Available in PMC 2021 Jun 7. (PMC7610920; doi:10.1017/S2045796021000342)
Supplement: Supplementary file 1 [file EMS124344-supplement-Supplementary_Material.docx]

# Supplementary Materials 

### Additional Details on Methods and Analysis

#### Additional Information on Categories of Marital Status as a Prognostic indicator

1) The included studies that provided individual patient data had marital status recorded in five categories: 1) married or cohabiting; 2) single (never married), 3) separated, 4) divorced, 5) widowed. However, some of the studies had very few participants that were divorced (n=0 in ITAS) and widowed (n=4 in GENPOD, n=1 in IPCRESS and n=6 in TREAD). Therefore the marital status variable was recoded into three categories in order to meet power requirements: i) married/cohabiting; ii) single; and iii) no longer married (this will include those that report being divorced, separated, or widowed). 

### Missing Data

Missing data were imputed using multiple imputation with chained equations (MICE) in [Stata](https://www.stata.com/) 16.0. Data not reasonably able to be log transformed to meet normality assumptions, were imputed using predictive mean matching (PMM) via a k-nearest neighbours approach as it is considered to be more appropriate for non-normal continuous variables (Horton and Lipsitz, 2001), here we used k=10. Linear regression was used for approximately normally distributed continuous variables, logistic regression models for binary variables, and ordinal and multinomial regression models for ordered and unordered categorical variables respectively.  All imputation models were built using data on baseline and outcome variables following conventions (Royston and White, 2011). Only variables with less than 50% missing data were imputed. All imputation models were run to produce 50 imputed datasets. 

### Software & Packages

Stata SE 16 (StataCorp, 2019): ipdmetan (Fisher, 2015), MICE (Royston, 2009), mi impute pmm (Morris, White and Royston, 2014) packages.

### Patient and Public Involvement

Service user advisory groups of two primary care mental health services in central London and the expert service user researchers of the Service User Research Forum (SURF) were consulted for advice on the design, conduct, and dissemination of this study.

## Details of preliminary searches, and additions, deviations and changes to protocols 

We registered the process of finding studies and the research questions for this study on PROSPERO (CRD42019129512) and produced a protocol paper which was amended twice. Below we explain the amendments made and the process of finding studies and forming the dataset for this study.

We started this project with one of the senior investigators (GL) in possession of individual patient data from two studies for which he was the chief investigator, and a third study that he was in the process of conducting. We ran preliminary searches informed by consultation with a librarian at University College London, to identify studies of depression in primary care using the MEDLINE database via OVID, hand-searching through reference lists of existing systematic reviews and contacting a number of experts to enquire about unpublished or ongoing studies. No limits or filters were applied to the searches and no automatic updates although searches were re-run as detailed below. From these searches it was noted that the CIS-R appeared to be the most commonly used comprehensive measures of depressive and anxiety symptoms and disorders in RCTs of depression set in primary care. Ten studies used the CIS-R at baseline to determine diagnosis (seven were published and three were protocols for trials that would be completed within a year or two of the searches), whereas one study set in primary care used the Schedules for Clinical Assessment in Neuropsychiatry (SCAN) (Wing *et al.*, 1990) and would likely have met all other inclusion criteria, although this is questionable as it was a partially randomized study only (Perroud *et al.*, 2012). In addition, only one study used the full Structured Clinical Interview for DSM (SCID) (Williams *et al.*, 1992) and would have met our other inclusion criteria (Hegerl *et al.*, 2010), see Supplementary Tables 5 and 6 for details. We therefore refined our preliminary searches to look for studies that used the CIS-R. That author (GL) was a co-investigator on a number of other trials that used the CIS-R and we made contact with the chief investigators of those studies to ask for in-principle agreement to access IPD from their trials. We then applied for funding for this project. Once funding was in place we registered our project on PROSPERO, at that point we had run two rounds of searches (preliminary searches and one set to inform our funding application), and we had obtained IPD data from four studies. We refined our searches by including two other databases and contacting experts for missed studies, this helped us find further studies. We invited the chief investigators from each of those studies to join the project. We began to collect some further IPD from the studies that had agreed to take part. We then wrote up a protocol paper with information of what we would do with those IPD data once the dataset was complete. We ran further searches and found one more study just before initially submitting the protocol paper. It was a protracted process to gain IPD from that study but the idea was that the Dep-GP IPD dataset would be formed from all of those studies we had found. The Protocol paper (including the searches) was peer-reviewed and we amended it post-review to give more details about this process. The protocol was then accepted for publication. It was amended once more when we decided that our choice of an I^2^ threshold for considering problematic heterogeneity was too high, we dropped it from 80% to 75% for all models and to 50% for the final models, in line with recommendations from Cochrane. We ran the final searches for studies meeting our inclusion criteria a few weeks before submitting this manuscript for publication and found no new studies meeting our criteria.

Our protocol paper provides information about all data we sought to extract from the included studies and all outcomes of interest. For the present study we were particularly interested in age, gender, and marital status, and potential confounders of the association between these factors and prognosis. We put together some exploratory directed acyclic graphs to consider what those confounders might be, and limited the data used for this study to those factors. Future studies using these data will consider the prognostic associations between other factors at baseline and prognosis. Further, for this study we amended our inclusion criteria slightly to exclude studies that did not include any measurement of age, gender, or marital status, or that sampled only those in one category of any of those variables. There were two changes to the statistical analysis plan that should be noted: we did not include attrition as an outcome for the present study, and we also did not include conversions of scored on depressive symptom scales to the PROMIS T-score (Choi *et al.*, 2014), this was because here we included one study with a scale that could not be converted to the PROMIS (the GHQ-12). 

## Additional References

**Balshem, H., Helfand, M., Schünemann, H. J., Oxman, A. D., Kunz, R., Brozek, J., Vist, G. E., Falck-Ytter, Y., Meerpohl, J. and Norris, S.** (2011) GRADE guidelines: 3. Rating the quality of evidence. *Journal of Clinical Epidemiology*, **64**, 401–406.

**Choi, S. W., Schalet, B. D., Cook, K. F. and Cella, D.** (2014) Establishing a common metric for depressive symptoms: Linking the BDI-II , CES-D, and PHQ-9 to PROMIS Depression. *Psychological Assessment*, **26**, 513–527.

**Cuijpers, P., Weitz, E., Twisk, J., Kuehner, C., Cristea, I., David, D., DeRubeis, R. J., Dimidjian, S., Miranda, J., Mohr, D. C., Segal, Z. V and Siddique, J.** (2014) Gender as predictor and moderator of outcome in cognitive behavior therapy and pharamcotherapy for adult depression: An "individual patient data” meta-analysis. *Depression and Anxiety*, **31**, 941–951.

**Fisher, D. J.** (2015) Two-stage individual participant data meta-analysis and generalized forest plots. *The Stata Journal*, **2**, 369–396.

**Guyatt, G. H., Oxman, A. D., Vist, G. E., Kunz, R., Falck-Ytter, Y., Alonso-Coello, P. and Schünemann, H. J.** (2008) GRADE: an emerging consensus on rating quality of evidence and strength of recommendations. *British Medical Journal (Clinical research ed.)*, **336**, 924–926.

**Hegerl, U., Hautzinger, M., Mergl, R., Kohnen, R., Schütze, M., Scheunemann, W., Allgaier, A.-K., Coyne, J. and Henkel, V.** (2010) Effects of pharmacotherapy and psychotherapy in depressed primary-care patients: a randomized, controlled trial including a patients’ choice arm. *The International Journal of Neuropsychopharmacology*, **13**, 31-44.

**Horton, N. J. and Lipsitz, S. R.** (2001) Multiple Imputation in Practice: Comparison of Software Packages for Regression Models With Missing Variables. *The American Statistician*, **55**, 244–254.

**Morris, T. P., White, I. R. and Royston, P.** (2014) Tuning multiple imputation by predictive mean matching and local residual draws. *BMC Medical Research Methodology*, **14**. Available at (https://bmcmedresmethodol.biomedcentral.com/track/pdf/10.1186/1471-2288-14-75.pdf). Accessed 4 May 2021.

**Perroud, N., Uher, R., Ng, M. Y. M., Guipponi, M., Hauser, J., Henigsberg, N., Maier, W., Mors, O., Gennarelli, M., Rietschel, M., Souery, D., Dernovsek, M. Z., Stamp, A. S., Lathrop, M., Farmer, A., Breen, G., Aitchison, K. J., Lewis, C. M., Craig, I. W. and McGuffin, P.** (2012) Genome-wide association study of increasing suicidal ideation during antidepressant treatment in the GENDEP project. *The Pharmacogenomics Journal*, **12**, 68–77.

**Royston, P.** (2009) Multiple imputation of missing values: Further update of ice, with an emphasis on categorical variables. *The Stata Journal*, 9, 466–477.

**Royston, P. and White, I. R.** (2011) Multiple Imputation by Chained Equations (MICE): Implementation in Stata. *Journal of Statistical Software*, **45**, 1–20.

**Siemieniuk, R. and Guyatt, G. H.** (2019) What is GRADE?. *BMJ best practice*. Available at (https://bestpractice.bmj.com/info/toolkit/learn-ebm/what-is-grade/). Accessed 4 May 2021. 

**StataCorp** (2019) Stata Statistical Software: Release 16. *Stata Statistical Software*. StataCorp LLC : College Station, TX.

**Weitz, E. S., Hollon, S. D., Twisk, J., Van Straten, A., Huibers, M. J. H., David, D., DeRubeis, R. J., Dimidjian, S., Dunlop, B. W., Cristea, I. A., Faramarzi, M., Hegerl, U., Jarrett, R. B., Kheirkhah, F., Kennedy, S. H., Mergl, R., Miranda, J., Mohr, D. C., Rush, A. J., Segal, Z. V., Siddique, J., Simons, A. D., Vittengl, J. R. and Cuijpers, P.** (2015) Baseline depression severity as moderator of depression outcomes between cognitive behavioral therapy vs pharmacotherapy: An individual patient data meta-analysis. *JAMA Psychiatry*, **72**, 1102–1109.

**Williams, J., Gibbon, M., First, M., Spitzer, R., Davies, M., Borus, J., Howes, M., Kane, J., Pope, H., Rounsaville, B. and Wittchen, H.** (1992) The structured clinical interview for DSM-III-R (SCID): II. Multisite test-retest reliability. *Archives of General Psychiatry*, **49**, 630–636.

**Wing, J. K., Barbor, T., Brugha, T. S., Burke, J., Cooper, J. E., Giel, R., Jablenski, A., Regier, D. A. and Sartorius, N.** (1990) SCAN. *Archives of General Psychiatry*, **47**, 589-593.

## Supplementary Tables 

### Details of search terms and search results for RCTs to form IPD dataset

**Supplementary Table 1.** Bibliographic database searches and results

| **Searches** | **Results** |
| --- | --- |
| **Cochrane CENTRAL Trial Register (searched on 1****st** **December 2020)** |  |
| 1. ("Depression" or "MDD" or "Unipolar" or "Depressive"):ti,ab,kw (Word variations have been searched) | 81260 |
| 2. (“RCT” or "controlled trial" or "randomized controlled trial" or "clinical trial"):ti,ab,kw (Word variations have been searched) | 1061566 |
| 3. ("CIS-R" or "Clinical Interview Schedule" or “Revised Clinical Interview Schedule” or “Clinical Interview Schedule Revised”):ti,ab,kw (Word variations have been searched) | 63 |
| 4. #1 and #2 and #3 | **49** |
| **Embase 1947 to 2020 November 30** |  |
| 1. (depression or Depressive disorder or Major depression or Unipolar depression or MDD).mp. | 7288455 |
| 2. exp controlled clinical trial/ or exp "randomized controlled trial (topic)"/ or exp "clinical trial"/ | 1757626 |
| 3. ("Clinical Interview Schedule" or "CIS-R" or "CISR" or "Revised clinical interview schedule" or "clinical interview schedule revised").af. | 857 |
| 4. 1 and 2 and 3 | **33** |
| **International Pharmaceutical Abstracts 1970 to October 2020** |  |
| 1. (depression or Depressive disorder or Major depression or Unipolar depression or MDD).mp. | 10409 |
| 2. (RCT or controlled trial or randomized controlled trial or clinical trial).mp. | 15679 |
| 3. ("Clinical Interview Schedule" or "CIS-R" or "CISR" or "Revised clinical interview schedule" or "clinical interview schedule revised").af. | 3 |
| 4. 1 and 2 and 3 | **0** |
| **Ovid MEDLINE 1946 to December  01 2020** |  |
| 1. exp major depression/ or exp "depression (emotion)"/ | 122064 |
| 2. exp Depressive Disorder, Major/ | 30626 |
| 3. exp Depressive Disorder, Major/ or exp Depressive Disorder/ or exp Depression/ | 219973 |
| 4. 1 or 2 or 3 | 219973 |
| 5. exp controlled clinical trial/ or exp "randomized controlled trial (topic)"/ | 608159 |
| 6. ("Clinical Interview Schedule" or "CIS-R" or "CISR" or "Revised clinical interview schedule" or "clinical interview schedule revised").af. | 621 |
| 7. 4 and 5 and 6 | **21** |
| **PsycINFO 1806 to November Week 4 2020** |  |
| 1. exp major depression/ or exp "depression (emotion)"/ | 158704 |
| 2. (depression or Depressive disorder or Major depression or Unipolar depression or MDD).mp. | 344487 |
| 3. 1 or 2 | 344715 |
| 4. exp "randomized controlled trial (topic)"/ or exp "clinical trial"/ or exp "controlled trial"/ or exp "randomized clinical trial"/ | 12545 |
| 5. (RCT or controlled trial or randomized controlled trial or clinical trial).mp. | 43921 |
| 6. 4 or 5 | 50453 |
| 7. ("Clinical Interview Schedule" or "CIS-R" or "CISR" or "Revised clinical interview schedule" or "clinical interview schedule revised").af. | 1223 |
| 8. 3 and 6 and 7 | **47** |

No limits or filters were set on searches. 

**Supplementary Table 2.** Ethical approval and Trial Registration details of the studies included in the Dep-GP IPD database

| **Study** | **Ethical Approvals** | **Trial Registration details** |
| --- | --- | --- |
| CADET | Granted by NHS Health Research Authority & NRES Committee South West (NRES/07/H1208/60) | ISRCTN32829227; https://doi.org/10.1186/ISRCTN32829227 |
| COBALT | Approvals were granted by West Midlands Research Ethics Committee (NRES/07/H1208/60) and research governance approval was obtained from the local Primary Care Trusts/Health Boards | ISRCTN38231611; https://doi.org/10.1186/ISRCTN38231611 |
| GENPOD | The South West Research Ethics Committee granted approval (MREC 02/6/076) and the Bristol, Manchester and Newcastle Primary Care NHS Trusts granted research governance approval. | ISRCTN31345163; https://doi.org/10.1186/ISRCTN31345163 |
| IPCRESS | Approval granted by Royal Free and Hampstead Research Ethics Committee, reference number 05/Q0501/18 | ISRCTN45444578; https://doi.org/10.1186/ISRCTN45444578 |
| ITAS | Bro Taf Health Authority and United Bristol Healthcare Trust Local Research Ethics Committee | ISRCTN57116180; https://doi.org/10.1186/ISRCTN57116180 |
| MIR | Approvals were granted by South East Wales Research Ethics Committee Panel C (ref: 12/WA/0353); Bristol Clinical Commissioning Group (CCG), and other CCGs provided research governance assurance. | ISRCTN06653773; https://doi.org/10.1186/ISRCTN06653773 |
| PANDA | The Bristol Research Ethics Committee Centre granted ethics approval (12/SW/0267). | ISRCTN84544741; https://doi.org/10.1186/ISRCTN84544741 |
| REEACT | The Leeds (East) research ethics committee granted approval (08/H1306/77). | ISRCTN91947481; https://doi.org/10.1186/ISRCTN91947481 |
| TREAD | Approvals were granted by West Midlands multicentre research ethics committee (MREC 05/MRE07/42), and research governance approval was given by the relevant local National Health Service primary care trusts | ISRCTN16900744; https://doi.org/10.1186/ISRCTN16900744 |

**Supplementary Table 3**. QUIPS risk of bias ratings

| **Study** | **Study Participation** | **Study Attrition** | **Prognostic Factor Measurement** | **Outcome Measurement** | **Study Confounding** | **Statistical Analysis and Reporting** |
| --- | --- | --- | --- | --- | --- | --- |
| CADET | Low | Moderate | Low | Low | Low | Low |
| COBALT | Low | Low | Low | Moderate | Low | Low |
| GENPOD | Low | Low | Low | Low | Low | Low |
| IPCRESS | Low | High | Low | Low | Low | Low |
| ITAS | Low | Low | Low | Low | Low | Low |
| MIR | Low | Moderate | Low | Low | Low | Low |
| PANDA | Low | Low | Low | Low | Low | Low |
| REEACT | Low | Moderate | Low | Low | Low | Low |
| TREAD | Low | Low | Low | Low | Low | Low |

**Supplementary Table 4.** GRADE quality rating for each evidence for each type prognostic factor assessed

|  |  | **CADET** | **COBALT** | **GENPOD** | **IPCRESS** | **ITAS** | **MIR** | **PANDA** | **REEACT** | **TREAD** | **Overall Rating** | **Overall Quality Per Prognostic Factor** |
| --- | --- | --- | --- | --- | --- | --- | --- | --- | --- | --- | --- | --- |
| **Age** | Risk of Bias | ++++ | ++++ | ++++ | ++++ | ++++ | ++++ | ++++ | ++++ | ++++ | ++++ | **HIGH** |
|  | Imprecision | ++++ | ++++ | ++++ | ++++ | ++++ | ++++ | ++++ | ++++ | ++++ | ++++ |  |
|  | Inconsistency | ++++ | ++++ | ++++ | ++++ | ++++ | ++++ | ++++ | ++++ | ++++ | ++++ |  |
|  | Indirectness | N/A | N/A | N/A | N/A | N/A | N/A | N/A | N/A | N/A | ++++ |  |
|  | Publication Bias | N/A | N/A | N/A | N/A | N/A | N/A | N/A | N/A | N/A | ++++ |  |
|  |  |  |  |  |  |  |  |  |  |  |  |  |
| **Gender** | Risk of Bias | ++++ | ++++ | ++++ | ++++ | ++++ | ++++ | ++++ | ++++ | ++++ | ++++ | **HIGH** |
|  | Imprecision | ++++ | ++++ | ++++ | ++++ | ++++ | ++++ | ++++ | ++++ | ++++ | ++++ |  |
|  | Inconsistency | ++++ | ++++ | ++++ | ++++ | ++++ | ++++ | ++++ | ++++ | ++++ | ++++ |  |
|  | Indirectness | N/A | N/A | N/A | N/A | N/A | N/A | N/A | N/A | N/A | ++++ |  |
|  | Publication Bias | N/A | N/A | N/A | N/A | N/A | N/A | N/A | N/A | N/A | ++++ |  |
|  |  |  |  |  |  |  |  |  |  |  |  |  |
| **Marital Status** | Risk of Bias | ++++ | ++++ | ++++ | ++++ | ++++ | ++++ | ++++ | ++++ | ++++ | ++++ | **HIGH** |
|  | Imprecision | ++++ | ++++ | ++++ | ++++ | ++++ | ++++ | ++++ | ++++ | ++++ | ++++ |  |
|  | Inconsistency | ++++ | ++++ | ++++ | ++++ | ++++ | ++++ | ++++ | ++++ | ++++ | ++++ |  |
|  | Indirectness | N/A | N/A | N/A | N/A | N/A | N/A | N/A | N/A | N/A | ++++ |  |
|  | Publication Bias | N/A | N/A | N/A | N/A | N/A | N/A | N/A | N/A | N/A | ++++ |  |
|  |  |  |  |  |  |  |  |  |  |  |  |  |
| **Overall Quality Per Study** |  | High | High | High | High | High | High | High | High | High |  |  |

These ratings were conducted independently by two reviewers, following recommendations (Guyatt *et al.*, 2008; Balshem *et al.*, 2011; Siemieniuk and Guyatt, 2019) this table combines the outcomes from both. Ratings were made for each prognostic factor within each included study, across each study as a whole, and for each prognostic factor across all included studies. Ratings of indirectness and publication bias were only considered applicable for the prognostic factors across all included studies, not within any individual study. 

**Supplementary Table 5.** Bibliographic database searches and results for Preliminary Searches

| **Searches** | **Results** |
| --- | --- |
| **Ovid MEDLINE 1860 to March 01 2016** |  |
| 1. exp major depression/ or exp "depression (emotion)"/ | 96427 |
| 2. exp Depressive Disorder, Major/ | 24516 |
| 3. exp Depressive Disorder, Major/ or exp Depressive Disorder/ or exp Depression/ | 183278 |
| 4. 1 or 2 or 3 | 183278 |
| 5. exp controlled clinical trial/ or exp "randomized controlled trial (topic)"/ | 529900 |
| 6. 4 and 5 | 14281 |
| 7. ("primary care" or "general practice" or "general practitioner pr GP").af. | 163285 |
| 8. "Schedules for Clinical Assessment in Neuropsychiatry".af. | 230 |
| 9. 6 and 7 and 8 | **2** |
| 10. ("structured clinical interview for DSM" or "SCID").af. | 29843 |
| 11. 6 and 7 and 10 | 19 |
| 12. ("Clinical Interview Schedule" or "CIS-R" or "CISR" or "Revised clinical interview schedule" or "clinical interview schedule revised").af. | 519 |
| 13. 6 and 7 and 12 | 12 |

**Supplementary Table 6.** Results from database searches and results for Preliminary Searches and reasons for exclusion

| **Notes on potential for inclusion** | **Title** | **Journal and Citation** | **How Found** | **First Author** |
| --- | --- | --- | --- | --- |
| Protocol | Stop or go? Preventive cognitive therapy with guided tapering of antidepressants during pregnancy: study protocol of a pragmatic multicentre non-inferiority randomized controlled trial. | BMC Psychiatry. 16:72, 2016 Mar 18. | MEDLINE | Molenaar, Nina M |
| Not RCT | Cost-effectiveness of active monitoring versus antidepressants for major depression in primary health care: a 12-month non-randomized controlled trial (INFAP study). | BMC Psychiatry. 15:63, 2015 Mar 31. | MEDLINE | Rubio-Valera, Maria |
| Only Depression Module of SCID | ACTIVEDEP: a randomised, controlled trial of a home-based exercise intervention to alleviate depression in middle-aged and older adults. | British Journal of Sports Medicine. 48(3):226-32, 2014 Feb. | MEDLINE | Pfaff, Jon J |
| Only Depression Module of SCID | Training primary health care workers in mental health and its impact on diagnoses of common mental disorders in primary care of a developing country, Malawi: a cluster-randomized controlled trial. | Psychological Medicine. 44(3):657-66, 2014 Feb. | MEDLINE | Kauye, F |
| Protocol | Tavistock Adult Depression Study (TADS): a randomised controlled trial of psychoanalytic psychotherapy for treatment-resistant/treatment-refractory forms of depression. | BMC Psychiatry. 12:60, 2012 Jun 11. | MEDLINE | Taylor, David |
| Protocol | Cost-effectiveness of nurse-led self-help for recurrent depression in the primary care setting: design of a pragmatic randomised controlled trial. | BMC Psychiatry. 12:59, 2012 Jun 07. | MEDLINE | Biesheuvel-Leliefeld, Karolien E M |
| Not Depression | A randomized, controlled clinical trial: the effect of mindfulness-based cognitive therapy on generalized anxiety disorder among Chinese community patients: protocol for a randomized trial. | BMC Psychiatry. 11:187, 2011 Nov 29. | MEDLINE | Wong, Samuel Y S |
| Protocol | Brief cognitive behavioral therapy compared to general practitioners care for depression in primary care: a randomized trial. | Trials [Electronic Resource]. 11:96, 2010 Oct 12. | MEDLINE | Baas, Kim D |
| Protocol | Community pharmacist intervention in depressed primary care patients (PRODEFAR study): randomized controlled trial protocol. | BMC Public Health. 9:284, 2009 Aug 05. | MEDLINE | Rubio-Valera, Maria |
| Not RCT | SPIFA-A presentation of the Structured Psychiatric Interview for General Practice. | Nordic Journal of Psychiatry. 63(6):443-53, 2009 Nov. | MEDLINE | Dahl, Alv A |
| Older Adults Only and not full SCID | Reducing suicidal ideation in depressed older primary care patients. | Journal of the American Geriatrics Society. 54(10):1550-6, 2006 Oct. | MEDLINE | Unutzer, Jurgen |
| Not RCT | Performance of the PHQ-9 as a screening tool for depression after stroke. | Stroke. 36(3):635-8, 2005 Mar. | MEDLINE | Williams, Linda S |
| Older Adults Only and not full SCID | Low yield of thyroid-stimulating hormone testing in elderly patients with depression. | General Hospital Psychiatry. 26(4):302-9, 2004 Jul-Aug. | MEDLINE | Fraser, Shelagh A |
| Only Depression Module of SCID | Controlled trial of the short- and long-term effect of psychological treatment of post-partum depression. I. Impact on maternal mood. | British Journal of Psychiatry. 182:412-9, 2003 May. | MEDLINE | Cooper, Peter J |
| Not RCT | Long-term prognosis of depression in primary care. | Bulletin of the World Health Organization. 78(4):439-45, 2000. | MEDLINE | Simon, G E |
| Only Depression Module of SCID | Recovery from depression, work productivity, and health care costs among primary care patients. | General Hospital Psychiatry. 22(3):153-62, 2000 May-Jun. | MEDLINE | Simon, G E |
| Personality Disorder study and SCID for Axis-II only | Personality disorder comorbidity with major depression and response to treatment with sertraline or citalopram. | International Clinical Psychopharmacology. 13(5):205-11, 1998 Sep. | MEDLINE | Ekselius, L |
| Not RCT | Depressive disorders in primary care: prevalence, functional disability, and identification. | Journal of General Internal Medicine. 10(1):7-12, 1995 Jan. | MEDLINE | Williams, J W Jr |
| Not RCT | Physical symptom attributions: a defining characteristic of somatoform disorders?. | General Hospital Psychiatry. 37(2):147-52, 2015 Mar-Apr. | MEDLINE | Frostholm, Lisbeth |
| Not Depression RCT | Does psychological treatment help only those patients with severe irritable bowel syndrome who also have a concurrent psychiatric disorder?. | Australian & New Zealand Journal of Psychiatry. 39(9):807-15, 2005 Sep. | MEDLINE | Creed, Francis |
| Protocol | Mirtazapine added to selective serotonin reuptake inhibitors for treatment-resistant depression in primary care (MIR trial): study protocol for a randomised controlled trial. | Trials [Electronic Resource]. 17:66, 2016 Feb 03. | MEDLINE | Tallon, Debbie |
| Meets all inclusion criteria | Clinical effectiveness of collaborative care for depression in UK primary care (CADET): cluster randomised controlled trial. | BMJ. 347:f4913, 2013 Aug 19. | MEDLINE | Richards, David A |
| Meets all inclusion criteria | Facilitated physical activity as a treatment for depressed adults: randomised controlled trial. | BMJ. 344:e2758, 2012 Jun 06. | MEDLINE | Chalder, Melanie |
| Not all in primary care | Economic evaluation of a task-shifting intervention for common mental disorders in India. | Bulletin of the World Health Organization. 90(11):813-21, 2012 Nov 01. | MEDLINE | Buttorff, Christine |
| Protocol | Cognitive behavioural therapy as an adjunct to pharmacotherapy for treatment resistant depression in primary care: the CoBalT randomised controlled trial protocol. | Contemporary Clinical Trials. 33(2):312-9, 2012 Mar. | MEDLINE | Thomas, Laura J |
| Protocol | Physical activity as a treatment for depression: the TREAD randomised trial protocol. | Trials [Electronic Resource]. 11:105, 2010 Nov 12. | MEDLINE | Baxter, Helen |
| Meets all inclusion criteria | The prevalence of suicidal ideation identified by the Edinburgh Postnatal Depression Scale in postpartum women in primary care: findings from the RESPOND trial. | BMC Pregnancy & Childbirth. 11:57, 2011 Aug 03. | MEDLINE | Howard, Louise M |
| Meets all inclusion criteria | A trial of problem-solving by community mental health nurses for anxiety, depression and life difficulties among general practice patients. The CPN-GP study. | Health Technology Assessment (Winchester, England). 9(37):1-104, iii, 2005 Sep. | MEDLINE | Kendrick, T |
| Not RCT | Factors associated with being a false positive on the General Health Questionnaire. | Social Psychiatry & Psychiatric Epidemiology. 40(5):402-7, 2005 May. | MEDLINE | Bell, Truda |
| Meets all inclusion criteria | A randomised controlled trial to compare the cost-effectiveness of tricyclic antidepressants, selective serotonin reuptake inhibitors and lofepramine. | Health Technology Assessment (Winchester, England). 9(16):1-134, iii, 2005 May. | MEDLINE | Peveler, R |
| Meets all inclusion criteria | Computerised patient-specific guidelines for management of common mental disorders in primary care: a randomised controlled trial. | British Journal of General Practice. 54(508):832-7, 2004 Nov. | MEDLINE | Thomas, Hollie V |
| Meets all inclusion criteria | Randomised controlled trial of problem solving treatment, antidepressant medication, and combined treatment for major depression in primary care. | BMJ. 320(7226):26-30, 2000 Jan 01. | MEDLINE | Mynors-Wallis, L M |
| Not study of MDD - excluded those with MDD | Effect of a Web-Based Guided Self-help Intervention for Prevention of Major Depression in Adults With Subthreshold Depression: A Randomized Clinical Trial. | JAMA. 315(17):1854-63, 2016 May 03. | Contact with Experts | Buntrock, Claudia |
| Pilot study only | Anticipate: A pilot randomised trial of CBT for antenatal depression and validation of depression screening by midwives. | Archives of Women's Mental Health | Contact with Experts | Evans J. |
| Protocol | A written self-help intervention for depressed adults comparing behavioural activation combined with physical activity promotion with a self-help intervention based upon behavioural activation alone: study protocol for a parallel group pilot randomised controlled trial (BAcPAc). | Trials. 15 (pp 196), 2014. Date of Publication: 2014. | Contact with Experts | Farrand P. |
| Feasibility trial only | Antidepressant and group psychosocial treatment for depression: a rater blind exploratory RCT from a low income country. | Behavioural & Cognitive Psychotherapy. 42(6):693-705, 2014 Nov. | Contact with Experts | Husain N |
| Not Primary Care | A controlled study of fluoxetine and cognitive-behavioural counselling in the treatment of postnatal depression. | BMJ (Clinical research ed.), 1997, 314(7085), 932-936. | Contact with Experts | Appleby L |
| Older Adults Only and Protocol | A randomised evaluation of CollAborative care and active surveillance for Screen-Positive EldeRs with sub-threshold depression (CASPER): study protocol for a randomized controlled trial | Trials, 2011, 12, 225-234. | Contact with Experts | Mitchell, Natasha |
| Older Adults Only and Protocol | CASPER plus (CollAborative care in Screen-Positive EldeRs with major depressive disorder): study protocol for a randomised controlled trial | Trials, 2014, 15(1), 451-463 | Contact with Experts | Overend, Karen |
| Not RCT | Outcomes of Depression International Network (ODIN) | British Journal of Psychiatry, 1998, 172(4), 359-363. | Contact with Experts | Dowrick, Christopher |
| Questionable for inclusion as only partially randomised - used full SCAN | Genome-wide association study of increasing suicidal ideation during antidepressant treatment in the GENDEP project | The Pharmacogenomics Journal, 2012, 12(1), 68-77. | Contact with Experts | Perroud, N |
| No comprehensive symptom or diagnostic screening measure; single self-report questionnaires only | Integrated primary care for patients with mental and physical multimorbidity: cluster randomised controlled trial of collaborative care for patients with depression comorbid with diabetes or cardiovascular disease | BMJ, 2015, 350:h638 | Contact with Experts | Coventry, Peter |
| Pilot study only | A pilot randomised controlled trial of cognitive behavioural therapy for antenatal depression | BMC Psychiatry. 13:33, 2013. | Contact with Experts | Burns A |
| Not Primary Care | Cognitive Therapy vs Medications in the Treatment of Moderate to Severe Depression | Arch Gen Psych, 2005, 62(4), 409-416. | Hand Searching Reference Lists | Robert J. DeRubeis |
| Not Primary Care | Randomized Trial of Behavioral Activation , Cognitive Therapy , and Antidepressant Medication in the Acute Treatment of Adults With Major Depression | JCCP, 2006, 74(4)., 658-670. | Hand Searching Reference Lists | Dimidjian, Sona |
| Not Primary Care | Depression beliefs, treatment preference, and outcomes in a randomized trial for major depressive disorder | J Psych Res, 2012, 46(3), 375-381. | Hand Searching Reference Lists | Dunlop, Boadie W |
| Not Primary Care | COGNITIVE THERAPY VERSUS FLUOXETINE IN THE TREATMENT OF DYSTHYMIC DISORDER | Depression, 1996, 41(4), 34-41. | Hand Searching Reference Lists | Dunner, David L |
| Not Primary Care | National Institute of Mental Health Treatment of Depression Collaborative Research Program General Effectiveness of Treatments | Arch Gen Psych, 1989, 46(11), 971-982. | Hand Searching Reference Lists | Irene Elkin |
| Used SCID data not presented but otherwise seems eligible | Effects of pharmacotherapy and psychotherapy in depressed primary-care patients: a randomized, controlled trial including a patients' choice arm | The International Journal of Neuropsychopharmacology, 2010, 13(1), 31 | Hand Searching Reference Lists | Hegerl, Ulrich |
| Not Primary Care and no comprehensive measure of symptoms and disorders | Cognitive Therapy and Pharamacotherapy for Depression Singly and in Combinatio | Arch Gen Psych, 1992, 49, 774-781 | Hand Searching Reference Lists | Hollon, Steven D. |
| Not Primary Care and no comprehensive measure of symptoms and disorders | Treatment of Atypical Depression With Cognitive Therapy or Phenelzine A Double-blind, Placebo-Controlled Trial | Arch Gen Psychiatry. 1999;56(5):431-437. | Hand Searching Reference Lists | Robin B. Jarrett |
| Not Primary Care, unclear if full SCID or just Depression Module | Differences in Brain Glucose Metabolism Between Responders to CBT and Venlafaxine in a 16-Week Randomized Controlled Trial Sidney | Am J Psychiatry 2007; 164:778–788 | Hand Searching Reference Lists | Kennedy, Sidney H |
| Not Primary Care, no comprehensive measure of symptoms and disorders | Miranda J, Chung JY, Green BL et al. Treating depression in pre- dominantly low-income young minority women: a randomized controlled trial. JAMA 2003;290:57-65. 68. | JAMA, 2003, 290(1), 57-65 | Hand Searching Reference Lists | Hegerl, Ulrich |
| Patients with Neurological Conditions | Comparative Outcomes for Individual Cognitive-Behavior Therapy, Supportive-Expressive Group Psychotherapy, and Sertraline for the Treatment of Depression in Multiple Sclerosis | JCCP, 2001, 69(6), 942-949 | Hand Searching Reference Lists | Mohr, David C. |
| Not Primary Care | Cognitive Therapy and Pharmacotherapy Singly and Together in the Treatment of Depression | Arch Gen Psychiatry. 1984;41(1):33-41 | Hand Searching Reference Lists | George E. Murphy, |
| Not Primary Care and unclear if data recorded from psychiatric interviews | Comparative Efficacy of Cognitive Therapy and Pharmacotherapy in the Treatment of Depressed Outpatients | Cognitive Therapy and Research, 1977, 1(1), 17-37. | Hand Searching Reference Lists | Rush, Augustus J |
| Not Primary Care and unclear if full SCID | Cognitive reactivity to sad mood provocation and the prediction of depressive relapse. | Arch Gen Psychiatry, 2006, 63(7), 749-755. | Hand Searching Reference Lists | Segal, Zindel V |

No limits or filters were set on searches, experts contacted included co-authors of this article and collaborators of theirs, hand searching of systematic reviews included (Cuijpers *et al.*, 2014; Weitz *et al.*, 2015). 

### Secondary Outcomes

**Supplementary Table 7.** Difference in Z-score of depressive symptoms (“mean difference”) at 6-8 months post-baseline per unit increase in baseline prognostic indicator.

|  | **Adjusted for treatment** |  |  | **Additionally adjusted for depressive severity and 'disorder characteristics'** |  |  | **Additionally adjusted for age, gender, and marital status** |  |  | **Additionally adjusted for employment status** |  |  |
| --- | --- | --- | --- | --- | --- | --- | --- | --- | --- | --- | --- | --- |
| **Baseline Variable** | **Z-score of depressive symptoms** | **Number of Studies** | **Heterogeneity** | **Z-score of depressive symptoms** | **Number of Studies** | **Heterogeneity** | **Z-score of depressive symptoms** | **Number of Studies** | **Heterogeneity** | **Z-score of depressive symptoms** | **Number of Studies** | **Heterogeneity** |
|  | **Z-score of depressive symptoms** | **K** | **I**^**2**^ | **Z-score of depressive symptoms** | **K** | **I**^**2**^ | **Z-score of depressive symptoms** | **K** | **I**^**2**^ | **Z-score of depressive symptoms** | **K** | **I**^**2**^ |
| Age (per 5 year increase) | -0.02(-0.04 to 0.01) | 5 | 37 | 0(-0.02 to 0.01) | 5 | 0 | -0.06(-0.20 to 0.08) | 5 | 0 | -0.07(-0.21 to 0.08) | 5 | 0 |
| Age Group⸷ | -0.04(-0.08 to 0.01) | 5 | 31 | -0.01(-0.05 to 0.02) | 5 | 0 | -0.11(-0.24 to 0.02) | 5 | 0 | -0.13(-0.26 to 0.01) | 5 | 0 |
| Age (16-29 - reference) | 0 |  |  | 0 |  |  | 0 |  |  | 0 |  |  |
| Age (30-39) | -0.07(-0.22 to 0.08) | 5 | 0 | -0.03(-0.13 to 0.07) | 5 | 13 | 0.00(-0.11 to 0.11) | 5 | 7 | -0.01(-0.11 to 0.09) | 5 | 0 |
| Age (40-49) | 0.08(-0.10 to 0.25) | 5 | 22 | 0.01(-0.13 to 0.16) | 5 | 55 | 0.04(-0.10 to 0.19) | 5 | 47 | 0.03(-0.12 to 0.18) | 5 | 49 |
| Age (50-59) | -0.09(-0.31 to 0.13) | 5 | 44 | 0.07(-0.08 to 0.21) | 5 | 49 | 0.08(-0.06 to 0.23) | 5 | 33 | 0.06(-0.08 to 0.19) | 5 | 25 |
| Age (60+) | -0.19(-0.43 to 0.05) | 5 | 44 | 0.05(-0.11 to 0.20) | 5 | 39 | 0.06(-0.11 to 0.24) | 5 | 40 | 0.05(-0.13 to 0.23) | 5 | 46 |
| Gender (women - reference) | 0 |  |  | 0 |  |  | 0 |  |  | 0 |  |  |
| Gender (men) | 0.15(0.05 to 0.25) | 5 | 0 | 0.16(0.06 to 0.25) | 5 | 6 | 0.16(0.06 to 0.26) | 5 | 10 | 0.13(0.03 to 0.23) | 5 | 14 |
| Marital Status⸷ | 0.11(0.06 to 0.17) | 5 | 0 | 0.07(0.02 to 0.13) | 5 | 0 | 0.08(0.02 to 0.13) | 5 | 0 | 0.06(0 to 0.11) | 5 | 0 |
| Married (reference) | 0 |  |  | 0 |  |  | 0 |  |  | 0 |  |  |
| Single | 0.15(0.04 to 0.27) | 5 | 0 | 0.10(-0.01 to 0.20) | 5 | 0 | 0.09(-0.02 to 0.21) | 5 | 0 | 0.05(-0.07 to 0.17) | 5 | 0 |
| No longer Married | 0.22(0.10 to 0.34) | 5 | 0 | 0.18(0.10 to 0.26) | 5 | 0 | 0.16(0.04 to 0.27) | 5 | 0 | 0.11(0.01 to 0.23) | 5 | 0 |
| Note: ⸷Association for ordinal variables is per category increase from first category shown below the variable down to the last (i.e. married to single, single to no longer married. ‘Disorder characteristics' adjusted for are: baseline BDI-II score, average anxiety duration, depression duration, comorbid panic disorder, and history of antidepressant treatment |  |  |  |  |  |  |  |  |  |  |  |  |

**Supplementary Table 8.** Percentage difference (“% difference”) in depressive symptom scale scores at 6-8 months post-baseline per unit increase in baseline prognostic indicator.

|  | **Adjusted for treatment** |  |  | **Additionally adjusted for depressive severity and 'disorder characteristics'** |  |  | **Additionally adjusted for age, gender, and marital status** |  |  | **Additionally adjusted for employment status** |  |  |
| --- | --- | --- | --- | --- | --- | --- | --- | --- | --- | --- | --- | --- |
| **Baseline Variable** | **Percentage difference in depressive symptoms** | **Number of Studies** | **Heterogeneity** | **Percentage difference in depressive symptoms** | **Number of Studies** | **Heterogeneity** | **Percentage difference in depressive symptoms** | **Number of Studies** | **Heterogeneity** | **Percentage difference in depressive symptoms** | **Number of Studies** | **Heterogeneity** |
|  | **% difference (95%CI)** | **K** | **I**^**2**^ | **% difference (95%CI)** | **K** | **I**^**2**^ | **% difference (95%CI)** | **K** | **I**^**2**^ | **% difference (95%CI)** | **K** | **I**^**2**^ |
| Age (per 5 year increase) | -0.99(-2.59 to 0.64) | 5 | 0 | -0.20(-1.65 to 1.26) | 5 | 15 | -6.59(-17.96 to 6.36) | 5 | 5 | -7.01(-17.95 to 5.38) | 5 | 0 |
| Age Group⸷ | -2.46(-5.69 to 0.88) | 5 | 13 | -0.84(-3.83 to 2.24) | 5 | 0 | -7.06(-17.17 to 4.28) | 5 | 0 | -8.1(-18.09 to 3.11) | 5 | 0 |
| Age (16-29 - reference) | 0 |  |  | 0 |  |  | 0 |  |  | 0 |  |  |
| Age (30-39) | -8.13(-19.67 to 5.06) | 5 | 0 | -7.87(-19.22 to 5.07) | 5 | 0 | -6.66(-18.81 to 7.31) | 5 | 0 | -6.71(-19.11 to 7.59) | 5 | 0 |
| Age (40-49) | 1.11(-15.16 to 20.51) | 5 | 41 | 1.77(-10.94 to 16.30) | 5 | 0 | 2.43(-12.34 to 19.70) | 5 | 13 | 0.81(-13.99 to 18.14) | 5 | 12 |
| Age (50-59) | 8.72(-23.83 to 9.40) | 5 | 39 | -3.90(-19.08 to 14.13) | 5 | 32 | -3.83(-21.02 to 17.11) | 5 | 37 | -6.09(-23.15 to 14.76) | 5 | 36 |
| Age (60+) | -14.98(-31.64 to 5.74) | 5 | 42 | -7.82(-20.95 to 7.48) | 5 | 0 | -9.36(-23.45 to 7.32) | 5 | 0 | -12.13(-28.00 to 7.25) | 5 | 15 |
| Gender (women - reference) | 0 |  |  | 0 |  |  | 0 |  |  | 0 |  |  |
| Gender (men) | 15.08(4.82 to 26.35) | 5 | 21 | 14.3(1.35 to 28.9) | 5 | 54 | 14.96(1.6 to 30.08) | 5 | 56 | 12.23(-1.69 to 28.13) | 5 | 62 |
| Marital Status⸷ | 7.69(2.69 to 12.94) | 5 | 0 | 5.09(0.36 to 10.03) | 5 | 0 | 5.41(0.68 to 10.36) | 5 | 0 | 3.5(-1.17 to 8.4) | 5 | 0 |
| Married (reference) | 0 |  |  | 0 |  |  | 0 |  |  | 0 |  |  |
| Single | 15.04(4.49 to 26.64) | 5 | 0 | 10.57(0.92 to 21.13) | 5 | 0 | 10.22(-0.06 to 21.55) | 5 | 0 | 6.47(-3.59 to 17.57) | 5 | 0 |
| No longer Married | 14.83(4.14 to 26.62) | 5 | 0 | 9.57(-0.33 to 20.46) | 5 | 0 | 10.13(0.14 to 21.11) | 5 | 0 | 6.54(-3.21 to 17.27) | 5 | 0 |
| Note: ⸷Association for ordinal variables is per category increase from first category shown below the variable down to the last (i.e. married to single, single to no longer married). ‘Disorder characteristics' adjusted for are: baseline BDI-II score, average anxiety duration, depression duration, comorbid panic disorder, and history of antidepressant treatment |  |  |  |  |  |  |  |  |  |  |  |  |

|  | **Adjusted for treatment** |  |  | **Additionally adjusted for depressive severity and 'disorder characteristics'** |  |  | **Additionally adjusted for age, gender, and marital status** |  |  | **Additionally adjusted for employment status** |  |  |
| --- | --- | --- | --- | --- | --- | --- | --- | --- | --- | --- | --- | --- |
| **Baseline Variable** | **Z-score of depressive symptoms** | **Number of Studies** | **Heterogeneity** | **Z-score of depressive symptoms** | **Number of Studies** | **Heterogeneity** | **Z-score of depressive symptoms** | **Number of Studies** | **Heterogeneity** | **Z-score of depressive symptoms** | **Number of Studies** | **Heterogeneity** |
|  | **Z-score of depressive symptoms** | **K** | **I**^**2**^ | **Z-score of depressive symptoms** | **K** | **I**^**2**^ | **Z-score of depressive symptoms** | **K** | **I**^**2**^ | **Z-score of depressive symptoms** | **K** | **I**^**2**^ |
| Age (per 5 year increase) | 0.01(-0.03 to 0.05) | 5 | 69 | 0.02(-0.01 to 0.04) | 5 | 62 | -0.09(-0.23 to 0.04) | 5 | 0 | -0.10(-0.23 to 0.03) | 5 | 0 |
| Age Group⸷ | 0.02(-0.06 to 0.09) | 5 | 59 | 0.04(-0.01 to 0.09) | 5 | 55 | -0.01(-0.14 to 0.11) | 5 | 0 | -0.01(-0.13 to 0.12) | 5 | 0 |
| Age (16-29 - reference) | 0 |  |  | 0 |  |  | 0 |  |  | 0 |  |  |
| Age (30-39) | 0.03(-0.12 to 0.17) | 5 | 0 | 0.04(-0.10 to 0.17) | 5 | 0 | 0.06(-0.08 to 0.19) | 5 | 0 | 0.04(-0.10 to 0.18) | 5 | 0 |
| Age (40-49) | 0.10(-0.12 to 0.33) | 5 | 61 | 0.05(-0.10 to 0.20) | 5 | 24 | 0.07(-0.13 to 0.27) | 5 | 50 | 0.05(-0.17 to 0.26) | 5 | 55 |
| Age (50-59) | 0.11(-0.15 to 0.37) | 5 | 65 | 0.15(0.01 to 0.29) | 5 | 0 | 0.15(-0.04 to 0.34) | 5 | 36 | 0.11(-0.09 to 0.31) | 5 | 39 |
| Age (60+) | 0.04(-0.34 to 0.41) | 5 | 80 | 0.17(-0.13 to 0.46) | 5 | 71 | 0.17(-0.19 to 0.53) | 5 | 77 | 0.09(-0.29 to 0.47) | 5 | 78 |
| Gender (women - reference) | 0 |  |  | 0 |  |  | 0 |  |  | 0 |  |  |
| Gender (men) | 0.1(-0.03 to 0.23) | 5 | 48 | 0.12(0.01 to 0.22) | 5 | 33 | 0.1(-0.01 to 0.22) | 5 | 36 | 0.08(-0.03 to 0.19) | 5 | 31 |
| Marital Status⸷ | 0.1(0.05 to 0.16) | 5 | 0 | 0.04(-0.01 to 0.1) | 5 | 0 | 0.04(-0.01 to 0.1) | 5 | 0 | 0.02(-0.03 to 0.07) | 5 | 0 |
| Married (reference) | 0 |  |  | 0 |  |  | 0 |  |  | 0 |  |  |
| Single | 0.13(0.03 to 0.24) | 5 | 0 | 0.07(-0.03 to 0.16) | 5 | 0 | 0.12(0.01 to 0.22) | 5 | 0 | 0.06(-0.05 to 0.17) | 5 | 0 |
| No longer Married | 0.20(0.09 to 0.32) | 5 | 0 | 0.09(-0.02 to 0.19) | 5 | 0 | 0.07(-0.04 to 0.18) | 5 | 0 | 0.03(-0.07 to 0.14) | 5 | 0 |
| Note: ⸷Association for ordinal variables is per category increase from first category shown below the variable down to the last (i.e. married to single, single to no longer married ‘Disorder characteristics' adjusted for are: baseline BDI-II score, average anxiety duration, depression duration, comorbid panic disorder, and history of antidepressant treatment |  |  |  |  |  |  |  |  |  |  |  |  |

**Supplementary Table 9.** Difference in Z-score of depressive symptoms (“mean difference”) at 9-12 months post-baseline per unit increase in baseline prognostic indicator.

**Supplementary Table 10.** Percentage difference (“% difference”) in depressive symptom scale scores at 9-12 months post-baseline per unit increase in baseline prognostic indicator.

|  | **Adjusted for treatment** |  |  | **Additionally adjusted for depressive severity and 'disorder characteristics'** |  |  | **Additionally adjusted for age, gender, and marital status** |  |  | **Additionally adjusted for employment status** |  |  |
| --- | --- | --- | --- | --- | --- | --- | --- | --- | --- | --- | --- | --- |
| **Baseline Variable** | **Percentage difference in depressive symptoms** | **Number of Studies** | **Heterogeneity** | **Percentage difference in depressive symptoms** | **Number of Studies** | **Heterogeneity** | **Percentage difference in depressive symptoms** | **Number of Studies** | **Heterogeneity** | **Percentage difference in depressive symptoms** | **Number of Studies** | **Heterogeneity** |
|  | **% difference (95%CI)** | **K** | **I**^**2**^ | **% difference (95%CI)** | **K** | **I**^**2**^ | **% difference (95%CI)** | **K** | **I**^**2**^ | **% difference (95%CI)** | **K** | **I**^**2**^ |
| Age (per 5 year increase) | 0.51(-2.63 to 3.74) | 4 | 69 | 1.80(0.14 to 3.50) | 4 | 0 | -6.89(-18.77 to 6.72) | 4 | 0 | -7.51(-19.21 to 5.88) | 4 | 0 |
| Age Group⸷ | 1.14(-4.88 to 7.54) | 4 | 66 | 3.79(0.35 to 7.34) | 4 | 0 | -1.41(-13.84 to 12.82) | 4 | 0 | -1.03(-13.46 to 13.19) | 4 | 0 |
| Age (16-29 - reference) | 0 |  |  | 0 |  |  | 0 |  |  | 0 |  |  |
| Age (30-39) | -1.62(-14.80 to 13.59) | 4 | 0 | -2.42(-15.10 to 12.14) | 4 | 0 | 0.46(-13.28 to 16.38) | 4 | 0 | -0.11(-13.86 to 15.83) | 4 | 0 |
| Age (40-49) | -0.13(-18.67 to 22.64) | 4 | 41 | -0.66(-12.91 to 13.31) | 4 | 0 | 3.82(-9.98 to 19.74) | 4 | 0 | 1.81(-14.42 to 21.12) | 4 | 26 |
| Age (50-59) | 3.47(-13.49 to 23.75) | 4 | 29 | 5.62(-8.66 to 22.14) | 4 | 0 | 9.50(-6.72 to 28.53) | 4 | 0 | 6.84(-9.10 to 25.58) | 4 | 0 |
| Age (60+) | 1.40(-28.09 to 42.98) | 4 | 74 | 16.38(-6.65 to 45.10) | 4 | 40 | 19.09(-9.92 to 57.44) | 4 | 56 | 6.81(-24.11 to 50.33) | 4 | 68 |
| Gender (women - reference) | 0 |  |  | 0 |  |  | 0 |  |  | 0 |  |  |
| Gender (men) | 11.09(-3.98 to 28.52) | 4 | 58 | 11.95(-3.09 to 29.33) | 4 | 60 | 11.4(-4.4 to 29.81) | 4 | 64 | 8.48(-6.81 to 26.29) | 4 | 63 |
| Marital Status⸷ | 8.08(1.05 to 15.6) | 4 | 34 | 3.64(-1.52 to 9.08) | 4 | 0 | 3.81(-1.37 to 9.27) | 4 | 0 | 1.58(-3.53 to 6.97) | 4 | 0 |
| Married (reference) | 0 |  |  | 0 |  |  | 0 |  |  | 0 |  |  |
| Single | 13.55(2.41 to 25.91) | 4 | 0 | 9.11(-1.26 to 20.57) | 4 | 0 | 15.82(4.20 to 28.73) | 4 | 0 | 9.69(-1.36 to 21.97) | 4 | 0 |
| No longer Married | 15.92(1.41 to 32.85) | 4 | 32 | 6.41(-4.24 to 18.24) | 4 | 0 | 4.56(-6.05 to 16.37) | 4 | 0 | 1.83(-8.62 to 13.47) | 4 | 0 |
| Note: ⸷Association for ordinal variables is per category increase from first category shown below the variable down to the last (i.e. married to single, single to no longer married). ‘Disorder characteristics' adjusted for are: baseline BDI-II score, average anxiety duration, depression duration, comorbid panic disorder, and history of antidepressant treatment |  |  |  |  |  |  |  |  |  |  |  |  |

**Supplementary Table 11.** Odds ratios for being in remission at 3-4 months post-baseline per unit increase in baseline prognostic indicator.

|  | **Adjusted for treatment** |  |  | **Additionally adjusted for depressive severity and 'disorder characteristics'** |  |  | **Additionally adjusted for age, gender, and marital status** |  |  | **Additionally adjusted for employment status** |  |  |
| --- | --- | --- | --- | --- | --- | --- | --- | --- | --- | --- | --- | --- |
| **Baseline Variable** | **Odds ratio for being in Remission at 3-4 months** | **Number of Studies** | **Heterogeneity** | **Odds ratio for being in Remission at 3-4 months** | **Number of Studies** | **Heterogeneity** | **Odds ratio for being in Remission at 3-4 months** | **Number of Studies** | **Heterogeneity** | **Odds ratio for being in Remission at 3-4 months** | **Number of Studies** | **Heterogeneity** |
|  | **OR(95%CI)** | **K** | **I**^**2**^ | **OR(95%CI)** | **K** | **I**^**2**^ | **OR(95%CI)** | **K** | **I**^**2**^ | **OR(95%CI)** | **K** | **I**^**2**^ |
| Age (per 5 year increase) | 1.02(0.98 to 1.07) | 8 | 65 | 1.00(0.95 to 1.05) | 8 | 54 | 1.03(0.79 to 1.33) | 8 | 0 | 1.03(0.79 to 1.33) | 8 | 0 |
| Age Group⸷ | 1.05(0.95 to 1.17) | 8 | 68 | 1.00(0.91 to 1.11) | 8 | 58 | 1.12(0.87 to 1.43) | 8 | 0 | 1.15(0.90 to 1.47) | 8 | 0 |
| Age (16-29 - reference) | 0 |  |  | 0 |  |  | 0 |  |  | 0 |  |  |
| Age (30-39) | 1.03(0.98 to 1.08) | 8 | 0 | 1.01(0.97 to 1.06) | 8 | 0 | 1.00(0.95 to 1.05) | 8 | 0 | 1.01(0.95 to 1.06) | 8 | 0 |
| Age (40-49) | 1.02(0.92 to 1.12) | 8 | 76 | 1.01(0.94 to 1.09) | 8 | 54 | 1.00(0.93 to 1.08) | 8 | 51 | 1.00(0.92 to 1.08) | 8 | 51 |
| Age (50-59) | 1.01(0.96 to 1.07) | 8 | 18 | 1.00(0.94 to 1.06) | 8 | 29 | 0.99(0.93 to 1.05) | 8 | 14 | 0.99(0.94 to 1.05) | 8 | 0 |
| Age (60+) | 1.07(0.96 to 1.19) | 8 | 55 | 1.01(0.92 to 1.10) | 8 | 50 | 0.99(0.90 to 1.10) | 8 | 48 | 1.00(0.91 to 1.10) | 8 | 43 |
| Gender (women - reference) | 0 |  |  | 0 |  |  | 0 |  |  | 0 |  |  |
| Gender (men) | 0.96(0.83 to 1.11) | 8 | 0 | 0.93(0.79 to 1.09) | 8 | 0 | 0.92(0.78 to 1.08) | 8 | 0 | 0.95(0.80 to 1.12) | 8 | 0 |
| Marital Status⸷ | 0.79(0.73 to 0.87) | 8 | 0 | 0.84(0.76 to 0.93) | 8 | 0 | 0.84(0.76 to 0.93) | 8 | 0 | 0.87(0.79 to 0.96) | 8 | 0 |
| Married (reference) | 0 |  |  | 0 |  |  | 0 |  |  | 0 |  |  |
| Single | 0.91(0.88 to 0.95) | 8 | 0 | 0.94(0.91 to 0.98) | 8 | 0 | 0.94(0.91 to 0.98) | 8 | 0 | 0.98(0.93 to 1.02) | 8 | 0 |
| No longer Married | 0.91(0.87 to 0.95) | 8 | 0 | 0.94(0.90 to 0.98) | 8 | 0 | 0.94(0.91 to 0.98) | 8 | 0 | 0.96(0.91 to 1.00) | 8 | 0 |
| Note: ⸷Association for ordinal variables is per category increase from first category shown below the variable down to the last (i.e. married to single, single to no longer married). ‘Disorder characteristics' adjusted for are: baseline BDI-II score, average anxiety duration, depression duration, comorbid panic disorder, and history of antidepressant treatment |  |  |  |  |  |  |  |  |  |  |  |  |

	

### Sensitivity Analyses

	

|  | **Adjusted for treatment, ‘disorder characteristics’, age, gender, marital status, employment status, and housing status^** |  |  | **^ and additionally adjusted for Long-term health condition status** |  |  | **^ and additionally adjusted for highest level of educational attainment** |  |  | **Adjusted for treatment, 'disorder characteristics', age, gender, marital status, employment status, and financial wellbeing**^**§**^ |  |  | **§ and additionally adjusted social support** |  |  |
| --- | --- | --- | --- | --- | --- | --- | --- | --- | --- | --- | --- | --- | --- | --- | --- |
| **Baseline Variable** | **Z-score of depressive symptoms** | **Number of Studies** | **Heterogeneity** | **Z-score of depressive symptoms** | **Number of Studies** | **Heterogeneity** | **Z-score of depressive symptoms** | **Number of Studies** | **Heterogeneity** | **Z-score of depressive symptoms** | **Number of Studies** | **Heterogeneity** | **Z-score of depressive symptoms** | **Number of Studies** | **Heterogeneity** |
|  | **Mean difference (95%CI)** | **K** | **I**^**2**^ | **Mean difference (95%CI)** | **K** | **I**^**2**^ | **Mean difference (95%CI)** | **K** | **I**^**2**^ | **Mean difference (95%CI)** | **K** | **I**^**2**^ | **Mean difference (95%CI)** | **K** | **I**^**2**^ |
| Age (per 5 year increase) | -0.06(-0.17 to 0.05) | 7 | 0 | -0.06(-0.17 to 0.05) | 7 | 0 | -0.04(-0.16 to 0.08) | 6 | 0 | -0.06(-0.18 to 0.06) | 6 | 0 | -0.06(-0.18 to 0.06) | 6 | 0 |
| Age Group⸷ | -0.01(-0.11 to 0.09) | 7 | 0 | -0.01(-0.11 to 0.09) | 7 | 0 | -0.02(-0.13 to 0.09) | 6 | 0 | -0.07(-0.18 to 0.04) | 6 | 0 | -0.06(-0.18 to 0.05) | 6 | 0 |
| Age (16-29 - reference) | 0 |  |  | 0 |  |  | 0 |  |  | 0 |  |  | 0 |  |  |
| Age (30-39) | -0.03(-0.10 to 0.16) | 7 | 24 | 0.02(-0.10 to 0.15) | 7 | 24 | -0.01(-0.15 to 0.14) | 6 | 30 | -0.12(-0.28 to 0.04) | 6 | 0 | -0.12(-0.28 to 0.04) | 6 | 0 |
| Age (40-49) | 0.07(-0.09 to 0.24) | 7 | 52 | 0.06(-0.11 to 0.23) | 7 | 52 | 0.00(-0.16 to 0.16) | 6 | 36 | -0.09(-0.25 to 0.23) | 6 | 0 | -0.10(-0.31 to 0.11) | 6 | 32 |
| Age (50-59) | 0.13(-0.03 to 0.29) | 7 | 34 | 0.10(-0.06 to 0.26) | 7 | 35 | 0.04(-0.11 to 0.19) | 6 | 14 | -0.02(-0.26 to 0.22) | 6 | 38 | 0.32(-0.03 to 0.21) | 6 | 40 |
| Age (60+) | 0.16(-0.03 to 0.36) | 7 | 41 | 0.12(-0.06 to 0.30) | 7 | 27 | 0.02(-0.14 to 0.19) | 6 | 0 | -0.07(-0.29 to 0.154) | 6 | 0 | -0.08(-0.30 to 0.14) | 6 | 0 |
| Gender (women - reference) | 0 |  |  | 0 |  |  | 0 |  |  | 0 |  |  | 0 |  |  |
| Gender (men) | 0.02(-0.05 to 0.09) | 7 | 0 | 0.02(-0.05 to 0.09) | 7 | 0 | 0.06(-0.02 to 0.13) | 6 | 0 | 0.02(-0.07 to 0.1) | 6 | 11 | 0.01(-0.07 to 0.09) | 6 | 11 |
| Marital Status⸷ | 0.06(0.01 to 0.1) | 7 | 0 | 0.06(0.02 to 0.1) | 7 | 0 | 0.06(0.01 to 0.11) | 6 | 0 | 0.06(0.02 to 0.11) | 6 | 0 | 0.06(0.01 to 0.11) | 6 | 0 |
| Married (reference) | 0 |  |  | 0 |  |  | 0 |  |  | 0 |  |  | 0 |  |  |
| Single | 0.05(-0.03 to 0.14) | 7 | 0 | 0.06(-0.03 to 0.14) | 7 | 0 | 0.07(-0.03 to 0.16) | 6 | 0 | 0.10(0.01 to 0.19) | 6 | 0 | 0.09(0.00 to 0.18) | 6 | 0 |
| No longer Married | 0.10(0.01 to 0.19) | 7 | 0 | 0.11(0.02 to 0.20) | 7 | 0 | 0.11(0.01 to 0.21) | 6 | 0 | 0.10(0.00 to 0.20) | 6 | 0 | 0.10(0.00 to 0.19) | 6 | 0 |
| Note:⸷Association for ordinal variables is per category increase from first category shown below the variable down to the last (i.e. married to single, single to no longer married). ‘Disorder characteristics' adjusted for are: baseline BDI-II score, average anxiety duration, depression duration, comorbid panic disorder, and history of antidepressant treatment. ^Adjusted for treatment, 'disorder characteristics', age, gender, marital status, employment status, and housing status. **§** Adjusted for treatment, 'disorder characteristics', age, gender, marital status, employment status, and financial wellbeing |  |  |  |  |  |  |  |  |  |  |  |  |  |  |  |

**Supplementary Table 12.** Difference in Z-score of depressive symptoms (“mean difference”) at 3-4 months post-baseline per unit increase in baseline prognostic indicator, adjusting for variables that were systematically missing in some studies.

|  | **Adjusted for treatment, 'disorder characteristics', age, gender, marital status, employment status, and housing status^** |  |  | **^ and additionally adjusted for Long-term health condition status** |  |  | **^ and additionally adjusted for highest level of educational attainment** |  |  | **Adjusted for treatment, 'disorder characteristics', age, gender, marital status, employment status, and financial wellbeing**^**§**^ |  |  | **§ and additionally adjusted social support** |  |  |
| --- | --- | --- | --- | --- | --- | --- | --- | --- | --- | --- | --- | --- | --- | --- | --- |
| **Baseline Variable** | **Percentage difference in depressive symptoms** | **Number of Studies** | **Heterogeneity** | **Percentage difference in depressive symptoms** | **Number of Studies** | **Heterogeneity** | **Percentage difference in depressive symptoms** | **Number of Studies** | **Heterogeneity** | **Percentage difference in depressive symptoms** | **Number of Studies** | **Heterogeneity** | **Percentage difference in depressive symptoms** | **Number of Studies** | **Heterogeneity** |
|  | **% difference (95%CI)** | **K** | **I**^**2**^ | **% difference (95%CI)** | **K** | **I**^**2**^ | **% difference (95%CI)** | **K** | **I**^**2**^ | **% difference (95%CI)** | **K** | **I**^**2**^ | **% difference (95%CI)** | **K** | **I**^**2**^ |
| Age (per 5 year increase) | -3.34(-1.87 to 6.02) | 7 | 0 | -3.34(-11.86 to 6.01) | 7 | 0 | -2.25(-11.47 to 7.93) | 6 | 0 | -2.20(-11.17 to 7.68) | 6 | 0 | -2.39(-11.38 to 7.52) | 6 | 0 |
| Age Group⸷ | -0.05(-8.34 to 8.98) | 7 | 0 | 0.15(-8.16 to 9.2) | 7 | 0 | 1.06(-7.93 to 10.93) | 6 | 0 | -5.25(-13.12 to 3.32) | 6 | 0 | -5(-12.89 to 3.6) | 6 | 0 |
| Age (16-29 - reference) | 0 |  |  | 0 |  |  | 0 |  |  | 0 |  |  | 0 |  |  |
| Age (30-39) | -1.31(-10.75 to 9.13) | 7 | 14 | -1.943(-11.40 to 8.53) | 7 | 15 | -4.54(-14.57 to 6.66) | 6 | 14 | -12.42(-23.35 to 0.07) | 6 | 0 | -12.63(-23.58 to -0.11) | 6 | 0 |
| Age (40-49) | 5.03(-6.59 to 18.11) | 7 | 31 | 4.24(-7.10 to 16.97) | 7 | 28 | 1.10(-9.61 to 13.07) | 6 | 12 | -7.07(-19.51 to 7.30) | 6 | 0 | -7.76(-20.15 to 6.55) | 6 | 0 |
| Age (50-59) | 10.32(-4.58 to 27.55) | 7 | 43 | 8.27(-6.13 to 24.88) | 7 | 39 | 4.72(-9.55 to 21.24) | 6 | 34 | 1.96(-20.84 to 31.31) | 6 | 59 | 1.48(-21.62 to 31.37) | 6 | 60 |
| Age (60+) | 13.07(-8.19 to 39.26) | 7 | 59 | 9.43(-9.62 to 32.50) | 7 | 49 | 2.84(-13.93 to 22.88) | 6 | 32 | -6.13(-27.93 to 22.26) | 6 | 39 | -6.18(-27.96 to 22.17) | 6 | 39 |
| Gender (women - reference) | 0 |  |  | 0 |  |  | 0 |  |  | 0 |  |  | 0 |  |  |
| Gender (men) | 2.75(-3.89 to 9.84) | 7 | 21 | 2.64(-4.18 to 9.96) | 7 | 26 | 5.52(-0.87 to 12.33) | 6 | 0 | 2.45(-5.87 to 11.52) | 6 | 44 | 1.99(-6.32 to 11.03) | 6 | 45 |
| Marital Status⸷ | 4.1(0.27 to 8.08) | 7 | 0 | 4.26(0.42 to 8.24) | 7 | 0 | 4.16(-0.16 to 8.67) | 6 | 6 | 4.55(0.74 to 8.5) | 6 | 0 | 4.27(0.46 to 8.21) | 6 | 0 |
| Married (reference) | 0 |  |  | 0 |  |  | 0 |  |  | 0 |  |  | 0 |  |  |
| Single | 6.20(-1.26 to 14.21) | 7 | 0 | 6.40(-1.07 to 14.45) | 7 | 0 | 7.63(-0.50 to 16.43) | 7 | 0 | 8.97(1.16 to 17.38) | 6 | 0 | 8.27(0.49 to 16.65) | 6 | 0 |
| No longer Married | 7.11(-0.87 to 15.74) | 7 | 0 | 7.39(-0.61 to 16.02) | 7 | 2 | 7.14(-1.63 to 16.68) | 7 | 2 | 7.31(-0.64 to 15.89) | 6 | 0 | 6.88(-1.04 to 15.42) | 6 | 0 |
| Note:⸷Association for ordinal variables is per category increase from first category shown below the variable down to the last (i.e. married to single, single to no longer married). ‘Disorder characteristics' adjusted for are: baseline BDI-II score, average anxiety duration, depression duration, comorbid panic disorder, and history of antidepressant treatment. ^Adjusted for treatment, 'disorder characteristics', age, gender, marital status, employment status, and housing status. **§** Adjusted for treatment, 'disorder characteristics', age, gender, marital status, employment status, and financial wellbeing |  |  |  |  |  |  |  |  |  |  |  |  |  |  |  |

**Supplementary Table 13.** Percentage difference (“% difference”) in depressive symptom scale scores at 3-4 months post-baseline per unit increase in baseline prognostic indicator, adjusting for variables that were systematically missing in some studies.

		

|  | **Adjusted for treatment, 'disorder characteristics', age, gender, marital status, employment status, and housing status^** |  |  | **^ and additionally adjusted for Long-term health condition status** |  |  | **^ and additionally adjusted for highest level of educational attainment** |  |  | **Adjusted for treatment, 'disorder characteristics', age, gender, marital status, employment status, and financial wellbeing**^**§**^ |  |  | **§ and additionally adjusted social support** |  |  |
| --- | --- | --- | --- | --- | --- | --- | --- | --- | --- | --- | --- | --- | --- | --- | --- |
| **Baseline Variable** | **Z-score of depressive symptoms** | **Number of Studies** | **Heterogeneity** | **Z-score of depressive symptoms** | **Number of Studies** | **Heterogeneity** | **Z-score of depressive symptoms** | **Number of Studies** | **Heterogeneity** | **Z-score of depressive symptoms** | **Number of Studies** | **Heterogeneity** | **Z-score of depressive symptoms** | **Number of Studies** | **Heterogeneity** |
|  | **Mean difference (95%CI)** | **K** | **I**^**2**^ | **Mean difference (95%CI)** | **K** | **I**^**2**^ | **Mean difference (95%CI)** | **K** | **I**^**2**^ | **Mean difference (95%CI)** | **K** | **I**^**2**^ | **Mean difference (95%CI)** | **K** | **I**^**2**^ |
| Age (per 5 year increase) | -0.11(-0.27 to 0.04) | 4 | 0 | -0.10(-0.26 to 0.06) | 4 | 0 | -0.07(-0.28 to 0.14) | 3 | 0 | -0.07(-0.21 to 0.07) | 5 | 0 | -0.05(-0.22 to 0.13) | 4 | 0 |
| Age Group⸷ | -0.13(-0.28 to 0.01) | 4 | 0 | -0.14(-0.28 to 0.01) | 4 | 0 | -0.14(-0.34 to 0.06) | 3 | 0 | -0.11(-0.25 to 0.02) | 5 | 0 | -0.11(-0.27 to 0.06) | 4 | 0 |
| Age (16-29 - reference) | 0 |  |  | 0 |  |  | 0 |  |  | 0 |  |  | 0 |  |  |
| Age (30-39) | -0.07(-0.24 to 0.11) | 4 | 0 | -0.0(-0.25 to 0.10) | 4 | 0 | -0.14(-0.36 to 0.08) | 3 | 0 | -0.14(-0.37 to 0.08) | 5 | 0 | -0.15(-0.28 to 0.04) | 4 | 0 |
| Age (40-49) | 0.07(-0.12 to 0.26) | 4 | 0 | 0.05(-0.14 to 0.24) | 4 | 0 | 0.00(-0.24 to 0.24) | 3 | 0 | -0.01(-0.25 to 0.23) | 5 | 0 | -0.01(-0.25 to 0.23) | 4 | 0 |
| Age (50-59) | -0.06(-0.26 to 0.14) | 4 | 0 | -0.08(-0.28 to 0.12) | 4 | 0 | -0.13(-0.40 to 0.13) | 3 | 0 | -0.14(-0.41 to 0.13) | 5 | 0 | -0.14(-0.41 to 0.13) | 4 | 0 |
| Age (60+) | -0.03(-0.26 to 0.19) | 4 | 0 | -0.06(-0.29 to 0.17) | 4 | 0 | -0.14(-0.45 to 0.18) | 3 | 0 | -0.15(-0.47 to 0.17) | 5 | 0 | -0.15(-0.47 to 0.17) | 4 | 0 |
| Gender (women - reference) | 0 |  |  | 0 |  |  | 0 |  |  | 0 |  |  | 0 |  |  |
| Gender (men) | 0.13(-0.01 to 0.26) | 4 | 37 | 0.13(0 to 0.27) | 4 | 38 | 0.15(-0.06 to 0.35) | 3 | 53 | 0.13(0.02 to 0.23) | 5 | 17 | 0.12(-0.02 to 0.27) | 4 | 39 |
| Marital Status⸷ | 0.04(-0.03 to 0.1) | 4 | 0 | 0.04(-0.03 to 0.1) | 4 | 0 | 0.04(-0.04 to 0.12) | 3 | 0 | 0.04(-0.02 to 0.09) | 5 | 0 | 0.04(-0.03 to 0.11) | 4 | 0 |
| Married (reference) | 0 |  |  | 0 |  |  | 0 |  |  | 0 |  |  | 0 |  |  |
| Single | 0.00(-0.14 to 0.14) | 4 | 0 | 0.01(-0.13 to 0.14) | 4 | 0 | 0.05(-0.13 to 0.22) | 3 | 0 | 0.04(-0.07 to 0.16) | 5 | 0 | 0.06(-0.08 to 0.19) | 4 | 0 |
| No longer Married | 0.08(-0.05 to 0.22) | 4 | 0 | 0.09(-0.05 to 0.22) | 4 | 0 | 0.08(-0.08 to 0.25) | 3 | 0 | 0.07(-0.05 to 0.19) | 5 | 0 | 0.06(-0.07 to 0.20) | 4 | 0 |
| Note:⸷Association for ordinal variables is per category increase from first category shown below the variable down to the last (i.e. married to single, single to no longer married). ‘Disorder characteristics' adjusted for are: baseline BDI-II score, average anxiety duration, depression duration, comorbid panic disorder, and history of antidepressant treatment. ^Adjusted for treatment, 'disorder characteristics', age, gender, marital status, employment status, and housing status. **§** Adjusted for treatment, 'disorder characteristics', age, gender, marital status, employment status, and financial wellbeing |  |  |  |  |  |  |  |  |  |  |  |  |  |  |  |

**Supplementary Table 14.** Difference in Z-score of depressive symptoms (“mean difference”) at 6-8 months post-baseline per unit increase in baseline prognostic indicator, adjusting for variables that were systematically missing in some studies.

**Supplementary Table 15.** Percentage difference (“% difference”) in depressive symptom scale scores at 6-8 months post-baseline per unit increase in baseline prognostic indicator, adjusting for variables that were systematically missing in some studies.

|  | **Adjusted for treatment, 'disorder characteristics', age, gender, marital status, employment status, and housing status^** |  |  | **^ and additionally adjusted for Long-term health condition status** |  |  | **^ and additionally adjusted for highest level of educational attainment** |  |  | **Adjusted for treatment, 'disorder characteristics', age, gender, marital status, employment status, and financial wellbeing**^**§**^ |  |  | **§ and additionally adjusted social support** |  |  |
| --- | --- | --- | --- | --- | --- | --- | --- | --- | --- | --- | --- | --- | --- | --- | --- |
| **Baseline Variable** | **Percentage difference in depressive symptoms** | **Number of Studies** | **Heterogeneity** | **Percentage difference in depressive symptoms** | **Number of Studies** | **Heterogeneity** | **Percentage difference in depressive symptoms** | **Number of Studies** | **Heterogeneity** | **Percentage difference in depressive symptoms** | **Number of Studies** | **Heterogeneity** | **Percentage difference in depressive symptoms** | **Number of Studies** | **Heterogeneity** |
|  | **% difference (95%CI)** | **K** | **I**^**2**^ | **% difference (95%CI)** | **K** | **I**^**2**^ | **% difference (95%CI)** | **K** | **I**^**2**^ | **% difference (95%CI)** | **K** | **I**^**2**^ | **% difference (95%CI)** | **K** | **I**^**2**^ |
| Age (per 5 year increase) | -13.60(-25.82 to 0.63) | 4 | 0 | -12.33(-24.69 to 2.05) | 4 | 0 | -8.28(-24.54 to 11.49) | 3 | 0 | -7.05(-17.99 to 5.35) | 5 | 0 | -3.85(-17.06 to 11.46) | 4 | 0 |
| Age Group⸷ | -9.44(-21.34 to 4.26) | 4 | 0 | -9.76(-21.57 to 3.83) | 4 | 0 | -9.07(-24.6 to 9.65) | 3 | 0 | -7.45(-17.49 to 3.82) | 5 | 0 | -6.51(-18.37 to 7.07) | 4 | 0 |
| Age (16-29 - reference) | 0 |  |  | 0 |  |  | 0 |  |  | 0 |  |  | 0 |  |  |
| Age (30-39) | -6.85(-21.00 to 9.13) | 4 | 14 | -7.41(-21.43 to 9.11) | 4 | 0 | -13.26(-29.40 to 6.56) | 3 | 0 | -13.70(-29.91 to 6.26) | 5 | 0 | -13.72(-29.99 to 6.33) | 4 | 0 |
| Age (40-49) | 4.13(-13.04 to 24.68) | 4 | 0 | 2.74(-14.20 to 23.03) | 4 | 0 | -4.21(-23.67 to 20.20) | 3 | 0 | -4.81(-24.33 to 19.75) | 5 | 0 | -4.77(-24.32 to 19.84) | 4 | 0 |
| Age (50-59) | -4.72(-27.60 to 25.39) | 4 | 48 | -5.27(-28.54 to 25.58) | 4 | 50 | -9.56(-41.32 to 39.12) | 3 | 64 | -10.18(-40.74 to 36.12) | 5 | 60 | -10.01(-41.49 to 38.41) | 4 | 63 |
| Age (60+) | -6.82(-32.59 to 28.80) | 4 | 42 | -7.26(-30.73 to 24.15) | 4 | 29 | -17.61(-50.76 to 37.87) | 3 | 54 | -19.43 (-52.16 to 35.68) | 5 | 55 | -19.28(-52.75 to 37.92) | 4 | 57 |
| Gender (women - reference) | 0 |  |  | 0 |  |  | 0 |  |  | 0 |  |  | 0 |  |  |
| Gender (men) | 13.54(-4.49 to 34.97) | 4 | 66 | 13.98(-4.26 to 35.69) | 4 | 66 | 15.4(-10.12 to 48.17) | 3 | 73 | 11.64(-2.4 to 27.7) | 5 | 63 | 11.34(-7.25 to 33.66) | 4 | 73 |
| Marital Status⸷ | 3.44(-2.68 to 9.95) | 4 | 0 | 3.52(-2.57 to 10) | 4 | 0 | 3.54(-4.02 to 11.7) | 3 | 0 | 2.18(-2.51 to 7.09) | 5 | 0 | 1.66(-3.68 to 7.29) | 4 | 0 |
| Married (reference) | 0 |  |  | 0 |  |  | 0 |  |  | 0 |  |  | 0 |  |  |
| Single | 2.92(-9.71 to 17.33) | 4 | 0 | 3.49(-9.19 to 17.93) | 4 | 0 | 9.42(-6.79 to 28.44) | 3 | 0 | 6.17(-3.86 to 17.25) | 5 | 0 | 7.35(-4.00 to 20.04) | 4 | 0 |
| No longer Married | 7.54(-5.15 to 21.93) | 4 | 0 | 7.57(-5.08 to 21.91) | 4 | 0 | 6.17(-9.08 to 23.96) | 3 | 0 | 3.55(-6.08 to 14.15) | 5 | 0 | 1.81(-8.86 to 13.74) | 4 | 0 |
| Note:⸷Association for ordinal variables is per category increase from first category shown below the variable down to the last (i.e. married to single, single to no longer married). ‘Disorder characteristics' adjusted for are: baseline BDI-II score, average anxiety duration, depression duration, comorbid panic disorder, and history of antidepressant treatment. ^Adjusted for treatment, 'disorder characteristics', age, gender, marital status, employment status, and housing status. **§** Adjusted for treatment, 'disorder characteristics', age, gender, marital status, employment status, and financial wellbeing |  |  |  |  |  |  |  |  |  |  |  |  |  |  |  |

|  | **Adjusted for treatment, 'disorder characteristics', age, gender, marital status, employment status, and housing status^** |  |  | **^ and additionally adjusted for Long-term health condition status** |  |  | **^ and additionally adjusted for highest level of educational attainment** |  |  | **Adjusted for treatment, 'disorder characteristics', age, gender, marital status, employment status, and financial wellbeing**^**§**^ |  |  | **§ and additionally adjusted social support** |  |  |
| --- | --- | --- | --- | --- | --- | --- | --- | --- | --- | --- | --- | --- | --- | --- | --- |
| **Baseline Variable** | **Z-score of depressive symptoms** | **Number of Studies** | **Heterogeneity** | **Z-score of depressive symptoms** | **Number of Studies** | **Heterogeneity** | **Z-score of depressive symptoms** | **Number of Studies** | **Heterogeneity** | **Z-score of depressive symptoms** | **Number of Studies** | **Heterogeneity** | **Z-score of depressive symptoms** | **Number of Studies** | **Heterogeneity** |
|  | **Mean difference (95%CI)** | **K** | **I**^**2**^ | **Mean difference (95%CI)** | **K** | **I**^**2**^ | **Mean difference (95%CI)** | **K** | **I**^**2**^ | **Mean difference (95%CI)** | **K** | **I**^**2**^ | **Mean difference (95%CI)** | **K** | **I**^**2**^ |
| Age (per 5 year increase) | -0.13(-0.28 to 0.02) | 4 | 0 | -0.13(-0.28 to 0.02) | 4 | 0 | -0.12(-0.27 to 0.03) | 4 | 0 | -0.07(-0.26 to 0.12) | 3 | 0 | -0.08(-0.27 to 0.11) | 3 | 0 |
| Age Group⸷ | 0.02(-0.12 to 0.15) | 4 | 0 | 0.02(-0.12 to 0.15) | 4 | 0 | 0(-0.14 to 0.14) | 4 | 0 | -0.03(-0.2 to 0.15) | 3 | 0 | -0.02(-0.19 to 0.16) | 3 | 0 |
| Age (16-29 - reference) | 0 |  |  | 0 |  |  | 0 |  |  | 0 |  |  | 0 |  |  |
| Age (30-39) | 0.02(-0.14 to 0.17) | 4 | 0 | 0.01(-0.14 to 0.17) | 4 | 0 | 0.01(-0.15 to 0.16) | 4 | 0 | -0.09(-0.37 to 0.20) | 3 | 0 | -0.09(-0.38 to 0.19) | 3 | 0 |
| Age (40-49) | 0.04(-0.12 to 0.20) | 4 | 0 | 0.03(-0.13 to 0.19) | 4 | 0 | 0.01(-0.15 to 0.17) | 4 | 0 | -0.15(-0.44 to 0.14) | 3 | 0 | -0.16(-0.44 to 0.13) | 3 | 0 |
| Age (50-59) | 0.11(-0.06 to 0.28) | 4 | 0 | 0.08(-0.09 to 0.26) | 4 | 0 | 0.05(-0.12 to 0.23) | 4 | 0 | -0.10(-0.40 to 0.20) | 3 | 0 | -0.10(-0.40 to 0.20) | 3 | 0 |
| Age (60+) | 0.08(-0.30 to 0.45) | 4 | 68 | 0.05(-0.28 to 0.39) | 4 | 59 | 0.01(-0.35 to 0.36) | 4 | 63 | -0.26(-0.62 to 0.09) | 3 | 0 | -0.26(-0.62 to 0.09) | 3 | 0 |
| Gender (women - reference) | 0 |  |  | 0 |  |  | 0 |  |  | 0 |  |  | 0 |  |  |
| Gender (men) | 0.08(-0.06 to 0.22) | 4 | 53 | 0.08(-0.06 to 0.22) | 4 | 52 | 0.08(-0.06 to 0.22) | 4 | 52 | 0.16(0.04 to 0.28) | 3 | 0 | 0.16(0.04 to 0.28) | 3 | 0 |
| Marital Status⸷ | 0.02(-0.05 to 0.08) | 4 | 0 | 0.02(-0.05 to 0.08) | 4 | 0 | 0.02(-0.04 to 0.08) | 4 | 0 | 0.02(-0.05 to 0.09) | 3 | 0 | 0.02(-0.05 to 0.09) | 3 | 0 |
| Married (reference) | 0 |  |  | 0 |  |  | 0 |  |  | 0 |  |  | 0 |  |  |
| Single | 0.00(-0.12 to 0.12) | 4 | 0 | 0.00(-0.12 to 0.12) | 4 | 0 | 0.01(-0.11 to 0.13) | 4 | 8 | 0.04(-0.10 to 0.19) | 3 | 0 | 0.05(-0.10 to 0.19) | 3 | 0 |
| No longer Married | 0.03(-0.10 to 0.15) | 4 | 0 | 0.03(-0.10 to 0.15) | 4 | 0 | 0.08(-0.08 to 0.25) | 4 | 0 | 0.03(-0.11 to 0.17) | 3 | 0 | 0.03(-0.11 to 0.17) | 3 | 0 |
| Note:⸷Association for ordinal variables is per category increase from first category shown below the variable down to the last (i.e. married to single, single to no longer married). ‘Disorder characteristics' adjusted for are: baseline BDI-II score, average anxiety duration, depression duration, comorbid panic disorder, and history of antidepressant treatment. ^Adjusted for treatment, 'disorder characteristics', age, gender, marital status, employment status, and housing status. **§** Adjusted for treatment, 'disorder characteristics', age, gender, marital status, employment status, and financial wellbeing |  |  |  |  |  |  |  |  |  |  |  |  |  |  |  |

**Supplementary Table 16.** Difference in Z-score of depressive symptoms (“mean difference”) at 9-12 months post-baseline per unit increase in baseline prognostic indicator, adjusting for variables that were systematically missing in some studies.

|  | **Adjusted for treatment, 'disorder characteristics', age, gender, marital status, employment status, and housing status^** |  |  | **^ and additionally adjusted for Long-term health condition status** |  |  | **^ and additionally adjusted for highest level of educational attainment** |  |  | **Adjusted for treatment, 'disorder characteristics', age, gender, marital status, employment status, and financial wellbeing**^**§**^ |  |  | **§ and additionally adjusted social support** |  |  |
| --- | --- | --- | --- | --- | --- | --- | --- | --- | --- | --- | --- | --- | --- | --- | --- |
| **Baseline Variable** | **Percentage difference in depressive symptoms** | **Number of Studies** | **Heterogeneity** | **Percentage difference in depressive symptoms** | **Number of Studies** | **Heterogeneity** | **Percentage difference in depressive symptoms** | **Number of Studies** | **Heterogeneity** | **Percentage difference in depressive symptoms** | **Number of Studies** | **Heterogeneity** | **Percentage difference in depressive symptoms** | **Number of Studies** | **Heterogeneity** |
|  | **% difference (95%CI)** | **K** | **I**^**2**^ | **% difference (95%CI)** | **K** | **I**^**2**^ | **% difference (95%CI)** | **K** | **I**^**2**^ | **% difference (95%CI)** | **K** | **I**^**2**^ | **% difference (95%CI)** | **K** | **I**^**2**^ |
| Age (per 5 year increase) | -13.49(-26.87 to 2.34) | 3 | 0 | -13.70(-27.05 to 2.09) | 3 | 0 | -13.54(-26.94 to 2.32) | 3 | 0 | -5.60(-20.84 to 12.58) | 3 | 0 | -6.21(-20.94 to 11.28) | 3 | 0 |
| Age Group⸷ | 0.78(-14.47 to 18.75) | 3 | 0 | 0.12(-15.04 to 17.99) | 3 | 0 | -0.54(-15.67 to 17.3) | 3 | 0 | -1.79(-16.43 to 15.41) | 3 | 0 | -1.25(-16.01 to 16.09) | 3 | 0 |
| Age (16-29 - reference) | 0 |  |  | 0 |  |  | 0 |  |  | 0 |  |  | 0 |  |  |
| Age (30-39) | -2.03(-17.59 to 16.47) | 3 | 0 | -7.41(-21.43 to 9.11) | 3 | 0 | -2.87(-18.38 to 15.58) | 3 | 0 | -10.83(-32.47 to 17.74) | 3 | 0 | -10.94(-32.62 to 17.70) | 3 | 0 |
| Age (40-49) | 0.91(-16.87 to 22.48) | 3 | 11 | 0.78(-15.67 to 20.43) | 3 | 0 | 0.71(-15.74 to 20.37) | 3 | 0 | -13.06(-34.60 to 15.58) | 3 | 0 | -13.18(-34.74 to 15.29) | 3 | 0 |
| Age (50-59) | 8.96(-10.97 to 33.34) | 3 | 41 | 5.82(-13.78 to 29.89) | 3 | 0 | 4.64(-14.85 to 28.62) | 3 | 0 | -0.38(-26.50 to 35.04) | 3 | 0 | -0.22(-26.44 to 35.34) | 3 | 0 |
| Age (60+) | 8.09(-31.32 to 70.10) | 3 | 68 | 5.75(-29.18 to 57.89) | 3 | 58 | 4.63(-29.46 to 55.19) | 3 | 57 | -17.58(-42.93 to 19.03) | 3 | 0 | -17.18(-42.81 to 19.94) | 3 | 0 |
| Gender (women - reference) | 0 |  |  | 0 |  |  | 0 |  |  | 0 |  |  | 0 |  |  |
| Gender (men) | 11.28(-11.5 to 39.92) | 3 | 74 | 11.44(-11.09 to 39.69) | 3 | 73 | 11.69(-10.74 to 39.75) | 3 | 72 | 14.9(-1.65 to 34.24) | 3 | 48 | 14.45(-2.14 to 33.87) | 3 | 49 |
| Marital Status⸷ | 3.69(-3.01 to 10.85) | 3 | 0 | 3.75(-2.95 to 10.9) | 3 | 0 | 3.69(-3.02 to 10.86) | 3 | 0 | 2.43(-4.8 to 10.22) | 3 | 26 | 2.18(-4.97 to 9.87) | 3 | 24 |
| Married (reference) | 0 |  |  | 0 |  |  | 0 |  |  | 0 |  |  | 0 |  |  |
| Single | 9.00(-5.00 to 25.06) | 3 | 0 | 8.80(-5.17 to 24.83) | 3 | 0 | 8.89(-5.14 to 25.00) | 3 | 0 | 8.86(-4.31 to 23.85) | 3 | 0 | 8.30(-4.78 to 23.18) | 3 | 0 |
| No longer Married | 5.54(-8.33 to 21.50) | 3 | 0 | 5.75(-8.12 to 21.70) | 3 | 0 | 5.68(-8.25 to 21.73) | 3 | 0 | 4.02(-10.84 to 21.34) | 3 | 28 | 3.63(-11.18 to 20.89) | 3 | 27 |
| Note:⸷Association for ordinal variables is per category increase from first category shown below the variable down to the last (i.e. married to single, single to no longer married). ‘Disorder characteristics' adjusted for are: baseline BDI-II score, average anxiety duration, depression duration, comorbid panic disorder, and history of antidepressant treatment. ^Adjusted for treatment, 'disorder characteristics', age, gender, marital status, employment status, and housing status. **§**  Adjusted for treatment, 'disorder characteristics', age, gender, marital status, employment status, and financial wellbeing |  |  |  |  |  |  |  |  |  |  |  |  |  |  |  |

**Supplementary Table 17.** Percentage difference (“% difference”) in depressive symptom scale scores at 9-12 months post-baseline per unit increase in baseline prognostic indicator, adjusting for variables that were systematically missing in some studies.

**Supplementary Table 18.** Odds ratios for being in remission at 3-4 months post-baseline per unit increase in baseline prognostic indicator, adjusting for variables that were systematically missing in some studies.

|  | **Adjusted for treatment, 'disorder characteristics', age, gender, marital status, employment status, and housing status^** |  |  | **^ and additionally adjusted for Long-term health condition status** |  |  | **^ and additionally adjusted for highest level of educational attainment** |  |  | **Adjusted for treatment, 'disorder characteristics', age, gender, marital status, employment status, and financial wellbeing**^**§**^ |  |  | **§ and additionally adjusted social support** |  |  |
| --- | --- | --- | --- | --- | --- | --- | --- | --- | --- | --- | --- | --- | --- | --- | --- |
| **Baseline Variable** | **Odds ratio for being in Remission at 3-4 months** | **Number of Studies** | **Heterogeneity** | **Odds ratio for being in Remission at 3-4 months** | **Number of Studies** | **Heterogeneity** | **Odds ratio for being in Remission at 3-4 months** | **Number of Studies** | **Heterogeneity** | **Odds ratio for being in Remission at 3-4 months** | **Number of Studies** | **Heterogeneity** | **Odds ratio for being in Remission at 3-4 months** | **Number of Studies** | **Heterogeneity** |
|  | **OR(95%CI)** | **K** | **I**^**2**^ | **OR(95%CI)** | **K** | **I**^**2**^ | **OR(95%CI)** | **K** | **I**^**2**^ | **OR(95%CI)** | **K** | **I**^**2**^ | **OR(95%CI)** | **K** | **I**^**2**^ |
| Age (per 5 year increase) | 1.03(0.78 to 1.36) | 7 | 0 | 1.04(0.79 to 1.37) | 7 | 0 | 0.99(0.73 to 1.35) | 6 | 0 | 1.03(0.75 to 1.41) | 6 | 0 | 1.04(0.76 to 1.42) | 6 | 0 |
| Age Group⸷ | 1.1(0.85 to 1.44) | 7 | 0 | 1.11(0.85 to 1.44) | 7 | 0 | 1.1(0.82 to 1.49) | 6 | 0 | 1.21(0.9 to 1.63) | 6 | 0 | 1.2(0.89 to 1.62) | 6 | 0 |
| Age (16-29 - reference) | 0 |  |  | 0 |  |  | 0 |  |  | 0 |  |  | 0 |  |  |
| Age (30-39) | 0.99(0.94 to 1.05) | 7 | 0 | 0.99(0.94 to 1.05) | 7 | 0 | 1.01(0.95 to 1.07) | 6 | 0 | 1.05(0.97 to 1.13) | 6 | 0 | 1.05(0.97 to 1.13) | 6 | 0 |
| Age (40-49) | 0.99(0.92 to 1.07) | 7 | 36 | 1.00(0.93 to 1.07) | 7 | 32 | 1.02(0.95 to 1.09) | 6 | 15 | 1.07(0.98 to 1.16) | 6 | 0 | 1.07(0.99 to 1.17) | 6 | 0 |
| Age (50-59) | 0.96(0.90 to 1.03) | 7 | 0 | 0.98(0.92 to 1.05) | 7 | 0 | 1.00(0.93 to 1.07) | 6 | 4 | 1.00(0.91 to 1.11) | 6 | 14 | 1.00(0.90 to 1.12) | 6 | 24 |
| Age (60+) | 0.96(0.86 to 1.06) | 7 | 39 | 0.98(0.90 to 1.08) | 7 | 22 | 1.01(0.93 to 1.11) | 6 | 0 | 1.07(0.96 to 1.20) | 6 | 0 | 1.07(0.96 to 1.20) | 6 | 0 |
| Gender (women - reference) | 0 |  |  | 0 |  |  | 0 |  |  | 0 |  |  | 0 |  |  |
| Gender (men) | 0.98(0.82 to 1.17) | 7 | 0 | 0.98(0.82 to 1.17) | 7 | 0 | 0.93(0.77 to 1.13) | 6 | 0 | 0.96(0.78 to 1.17) | 6 | 0 | 0.97(0.8 to 1.19) | 6 | 0 |
| Marital Status⸷ | 0.88(0.79 to 0.99) | 7 | 0 | 0.88(0.78 to 0.98) | 7 | 0 | 0.89(0.78 to 1) | 6 | 0 | 0.87(0.77 to 0.98) | 6 | 0 | 0.88(0.78 to 1) | 6 | 0 |
| Married (reference) | 0 |  |  | 0 |  |  | 0 |  |  | 0 |  |  | 0 |  |  |
| Single | 0.98(0.93 to 1.02) | 7 | 0 | 0.98(0.93 to 1.02) | 7 | 0 | 0.97(0.93 to 1.02) | 6 | 0 | 0.95(0.91 to 1.00) | 6 | 0 | 0.96(0.91 to 1.00) | 6 | 0 |
| No longer Married | 0.96(0.91 to 1.00) | 7 | 0 | 0.95(0.91 to 1.00) | 7 | 0 | 0.96(0.91 to 1.01) | 6 | 0 | 0.96(0.91 to 1.01) | 6 | 0 | 0.96(0.91 to 1.01) | 6 | 0 |
| Note:⸷Association for ordinal variables is per category increase from first category shown below the variable down to the last (i.e. married to single, single to no longer married).  ‘Disorder characteristics' adjusted for are: baseline BDI-II score, average anxiety duration, depression duration, comorbid panic disorder, and history of antidepressant treatment. ^Adjusted for treatment, 'disorder characteristics', age, gender, marital status, employment status, and housing status. **§** Adjusted for treatment, 'disorder characteristics', age, gender, marital status, employment status, and financial wellbeing |  |  |  |  |  |  |  |  |  |  |  |  |  |  |  |

**Supplementary Table 19.** Results of original analyses and corresponding sensitivity analyses.

| **Outcome Variable** | **Analysis** | **Pooled Effect Estimate - mean difference (95%CI)** |
| --- | --- | --- |
| z-score at 3-4 months | Original analysis of age group (40-49 compared to 16-29 years old) adjusted for treatment only | -0.00(-0.21 to 0.21) |
|  | Analysis removing study contributing most to heterogeneity (GENPOD) | -0.01(-0.26 to 0.24) |
| z-score at 9-12 months | Original analysis of age group (60+ compared to 16-29 years old)  adjusted for treatment only | -0.04(-0.34 to 0.41) |
|  | Analysis removing study contributing most to heterogeneity (TREAD and MIR) | -0.04(-0.64 to 0.18) |
| z-score at 9-12 months | Original analysis of age group (60+ compared to 16-29 years old)  adjusted for treatment, ‘disorder characteristics’, gender, and marital status | 0.17(-0.19 to 0.53) |
|  | Analysis removing study contributing most to heterogeneity (TREAD and MIR) | 0.39(-0.02 to 0.80) |
| z-score at 9-12 months | Original analysis of age group (60+ compared to 16-29 years old)  adjusted for treatment, ‘disorder characteristics’, gender, marital status, and employment status | 0.09(-0.29 to 0.47) |
|  | Analysis removing study contributing most to heterogeneity (TREAD and MIR) | 0.33(-0.04 to 0.70) |
| Remission at 3-4 months | Original analysis of age group (40-49 compared to 16-29 years old)  adjusted for treatment only | 1.02(0.92 to 1.12) |
|  | Analysis removing study contributing most to heterogeneity (GENPOD) | 1.04(0.99 to 1.10) |
|  |  |  |

### PRISMA-IPD Checklist
